# The Clinical Application of Bone Marrow Grafting

**DOI:** 10.1038/bjc.1962.47

**Published:** 1962-09

**Authors:** D. E. Pegg, J. G. Humble, K. A. Newton


### THE CLINICAL APPLICATION OF BONE MARROW GRAFTING

D. E. PEGG, J. G. HUMBLE AND K. A. NEWTON

*From the Departments of Haematology and Radiotherapy, Westminster Hospital and Medical School, London*

Received for publication June 15, 1962

It is now well recognised that bone marrow depression is often the limiting factor in the chemotherapy of malignant disease and that similar side effects may complicate large volume radiotherapy. It seemed logical to suppose that if these adverse effects could be modified or averted, then larger doses of anti-tumour drugs could be employed or large volume radiotherapy carried to higher levels of dosage. The well known effects of bone marrow cell infusion in laboratory animals suggested a possible attack on these problems in man.

It has been shown by the elegant experiments of Jacobson and his colleagues in mice (Jacobson *et al.*, 1949*a*, *b* ; 1950) that protection against the lethal effects of X-rays can be provided by splenic shielding. Similarly the aplasia produced by large doses of mustine hydrochloride was averted by clamping the splenic pedicle. Intra-peritoneal implantation of fresh splenic cells was also found to be protective and Lorenz and his colleagues (Lorenz *et al.*, 1951) obtained protection against X-ray aplasia with repopulation of bone marrow using intravascular injections of fresh bone marrow cells. Protection against lethal doses of alkylating agents was also provided by bone marrow cells injected intravenously (Weston *et al.*, 1957 ; Talbot and Elson, 1958 ; Tran Ba Loc, Mathé and Bernard, 1958 ; Dunjic and Maisin, 1960).

The clinical application of this animal work was facilitated by Barnes and Loutit (1955) when they showed that infant mouse spleen preserved at −79° C. by the cooling technique of Polge and Lovelock (1952) would protect irradiated mice. Thus it was theoretically possible to preserve part of a patient's own bone marrow and to reinfuse it later. This would obviate the necessity of using homologous bone marrow with its lower efficiency (Lorenz *et al.*, 1952), its danger of producing secondary disease, (Barnes and Loutit, 1954 ; Mathé *et al.*, 1960) and its ineffectiveness after alkylating agents (Talbot and Elson, 1958 ; Tran Ba Loc *et al.*, 1958). Many workers have attempted homologous, isologous and autologous bone marrow cell transplants in the human with varying degrees of success (Kay and Koller, 1960). Following similar principles we have treated 52 cases, 29 of whom received autologous marrow and 23 homologous marrow cell infusions.

### METHODS

(1) *Collection, storage and administration of bone marrow cells in the human.*— Preliminary clinical trials (Humble and Newton, 1958) showed that it was possible to aspirate 0·6–1·2 × $10^9$ cells by conventional bone marrow puncture and that such cell suspensions injected directly intravascularly seldom produced clinical symptoms. Early attempts to preserve aspirated marrow by the Polge-Smith-

Parkes technique were unsatisfactory. On cooling the cell suspension the fat formed a solid mass, trapping many of the bone marrow cells. This material could not be injected directly into the patient without risk of fat embolism, whereas filtration at this stage removed many of the bone marrow cells. Furthermore, apparatus available for this freezing technique was time consuming and inconvenient. Apparatus and techniques for the collection, processing, storage and administration of human bone marrow have already been described (Pegg and Trotman, 1959; Pegg, 1960; Pegg and Kemp, 1960).

(2) *Haematological Procedures.*—Standard haematological techniques were used throughout. Bone marrow cellularity was assessed by needle puncture withdrawing no more than 0·5 ml. with the preparation of smears and total nucleated cell counts. Myelograms were converted into absolute counts of each cell type. A normal range was established by biopsy of unirradiated bone marrow sites in 22 cases. The normal range was taken to be the mean $\pm$ two standard deviations. In describing the recovery of the peripheral blood counts of these patients we have used the term "beginning of recovery" to signify a rise in the count to twice its lowest value when this rise subsequently continued. The term "recovery" is reserved for restoration of the count to the normal range. Marrow cell infusion doses are given in terms of actual marrow cells corrected for leucocyte admixture.

### *Autologous Marrow Infusion in Wide Field Irradiation*

Eight patients received total thoracic radiotherapy for multiple pulmonary metastases, one patient received whole trunk irradiation for widely disseminated metastases and one patient received whole body irradiation.

### *Results*

The clinical and therapeutic details concerning these cases are shown in Table I. Cases 1 to 9 were treated by the 2 MeV Generator and Case 10 by X-rays generated at 250 kv. Cases 1, 2 and 4 received 300 r per day and developed severe radiation pneumonitis. The remainder of the patients receiving total thoracic irradiation were, therefore, given 140 to 150 r per day, but in spite of this, Case 7, a male of 52 with a history of chronic bronchitis, also developed radiation pneumonitis.

The pre-treatment blood counts revealed only minor departures from normality in these cases. Leucopenia followed treatment in Case 7 alone; this patient had previously received extensive radiotherapy to the pelvis and lumbar vertebrae.

In four cases (4 to 7 inclusive) it was possible to carry out a sternal marrow biopsy immediately after the course of radiotherapy and in each case this showed gross hypoplasia. Case 3 received an intravenous infusion of marrow freshly aspirated from his unirradiated pelvis, but the remaining patients were infused with marrow aspirated before treatment and stored at $-79°$ C. The amount of marrow infused varied between 3 and 133 $\times$ $10^6$ marrow cells per kg. Sternal marrow biopsies taken within 2–4 weeks of marrow infusion showed recovery to mild hypocellularity (Cases 1 and 5), or even to the lower limits of normal cellularity (Cases 2 and 7). Biopsies taken after 6–8 weeks usually showed complete marrow recovery (Cases 1, 6 and 7), although one patient (Case 5) had persistent mild hypocellularity. Case 9 received total trunk irradiation in a single treat-

TABLE I.—*Autologous Marrow Infusion in Wide Field Irradiation*

| Case | Age | Sex | Diagnosis | Total radiation dose (r) | Daily radiation dose (r) | Tumour regression | Radiation pneumonitis | Survival time | Cause of death |
|---|---|---|---|---|---|---|---|---|---|
| 1 | 21 | F. | Osteogenic sarcoma | 2708 | 300 | No | Yes | 13 weeks | Radiation pneumonitis |
| 2 | 21 | ,, | Ewings tumour | 2933 | 300 | Good | Yes | 7 months | Ditto |
| 3 | 49 | M. | Seminoma | 2500 | 150 | Fair | No | 16 days | Broncho-pneumonia |
| 4 | 19 | M. | Ewings tumour | 2950 | 300 | Good | Yes | 6 months | Radiation pneumonitis |
| 5 | 20 | ,, | Ditto | 3058 | 150 | ,, | No | 2 years | Pulmonary metastases |
| 6 | 5 | F. | Nephro-blastoma | 2550 | 150 | ,, | ,, | Alive at 3 years | — |
| 7 | 52 | M. | Seminoma | 2550 | 150 | Fair | Yes | 12 weeks | Radiation pneumonitis |
| 8 | 8 | ,, | Ewings tumour | 2550 | 140 | Good | No | 8 months | Cerebral metastases |
| 9 | 17 | ,, | Lympho-sarcoma | 560 | — | ,, | ,, | 9 weeks | Multiple metastases |
| 10 | 27 | ,, | Reticulum cell sarcoma | 300–350 | — | Fair | ,, | 20 days | Broncho-pneumonia |

ment session with minimal side effects. Half of his stored marrow was infused 4 days later, and the remaining half after a further 3 days. Recovery of his blood count began 9 days after the first infusion and was complete 9 days later. Case 10 was treated by Dr. B. Jolles and is included with his kind permission. Total body irradiation was administered in a single treatment session to a centre dose of 300–350 r and the bone marrow, stored at 4° C., was given 2 hr. later. He experienced a considerable leukopenia (Fig. 1), but recovery was rapid. The response of the tumour in these 10 patients followed their established radio-sensitivities.

### *Discussion*

In the human, regional irradiation of bone marrow frequently results in permanent hypoplasia of the treated area and this may be an important limiting factor in clinical radiotherapy. Assessment of the radiation dose absorbed by the bone marrow is a very complex problem, particularly when 250 kv radiation is used, and it is doubtful if accurate corrections can be made on a theoretical basis. Most of the authors specify radiation in skin doses, but several factors, including the superficial position of the marrow cavities sampled and secondary electron emission at 250 kv, make it rather unlikely that the bone marrow doses were less than 75 per cent of the skin doses.

Denstad (1943) found that complete recovery was unusual when the total skin dose, conventionally fractionated, exceeded 3000 r. Stewart and Dische (1956) and Stewart (1958) found that after 2000 r of fractionated radiation to the skin, recovery of the underlying bone marrow was not evident for 8–10 weeks and was often incomplete. After 4000 r aplasia was permanent in 50 per cent of cases and recovery in the remainder was slow and incomplete. The data of Sykes *et al.* (1959, 1960) confirm that the first signs of recovery after 4000 r are not usually seen until 2 months have elapsed and that complete eventual recovery is exceptional.

The dosage schedules used in standard radiotherapy procedures which involve irradiation of the sternum are quite different from those used in the present study, so that precise controls could not be obtained. Nevertheless it seems to be clear that the radiation doses used in these patients would have produced prolonged hypoplasia of the thoracic marrow in a high proportion of cases.

In 5 of our patients it was possible to obtain the marrow biopsies needed to establish the rate and extent of sternal marrow repopulation. Normal cellularity

FIG. 1.—Case 10.—Effect of autologous marrow infusion in a patient receiving whole body radiotherapy.

was achieved in 4–8 weeks in 4 of them ; the remaining case (No. 5), had some residual hypoplasia. It seems, therefore, that recovery was more rapid and more complete than would have occurred without marrow infusion.

It is well known that total haemopoiesis is not affected by local irradiation of quite large volumes (Denstad, 1943 ; Tubiana *et al.*, 1959). This is confirmed by the lack of leukopenia and thrombocytopenia in Cases 1–6 and 8 of the present series. Irradiation of the whole trunk or whole body, however, (Cases 9 and 10) did result in serious leukopenia. These patients recovered a normal white cell count in the remarkably short times of 18 and 14 days respectively. The time taken for complete recovery of the peripheral blood after 300–350 rads of total body irradiation is not altogether certain. The experience of accidental irradiation (Andrews *et al.*, 1959 ; Jammet *et al.*, 1959) suggests that 50–60 days might be a reasonable estimate. It seems probable, therefore, that the speedy recovery of our case was assisted by the marrow infusion. However, there are features of the accidental irradiation, particularly its lack of homogenicity and large neutron component, which suggest that the two situations may not be strictly comparable.

In this series it was not possible to demonstrate a relationship between the rapidity or extent of sternal marrow repopulation with the number of marrow cells infused ; 6 of the patients received more than the acceptable minimum autologous marrow cell infusion ($11 \times 10^6$ cells/kg., Pegg, 1962) ; the 4 patients who received less than this (Cases 2, 3, 5 and 7) had only thoracic irradiation. It is possible that less marrow is required to reseed locally aplased bone marrow than is needed for whole body radiation protection.

### *Autologous Marrow Infusion in Anti-tumour Chemotherapy*

Nineteen cases were studied.

#### *Results*

In all the patients in this study the malignant disease was in a very advanced state ; they were divided into six groups, each receiving a different alkylating agent. With each drug, either the speed of administration, or the total dose, or both, were increased above the usual treatment schedules (Table II).

*Group I. Mustine Hydrochloride* (5 *patients, Cases* 11–15).—The total doses were between two and four times as great as those commonly employed and the course lasted a very much shorter time than is usual. General toxic effects of the treatment were prominent and included severe nausea and vomiting, pulmonary oedema, hallucinations, deafness, pyrexia, diarrhoea and alopecia. Intravenous infusions of stored autologous bone marrow were given in all cases within 3 days of the completion of treatment, but in spite of this, all but 1 patient developed severe leukopenia and thrombocytopenia. One patient received 0·8 mg./kg. of mustine hydrochloride by lower limb perfusion, followed 7 hr. later by the intravenous infusion of autologous bone marrow stored at + 4° C. He suffered no more than a transient lymphopenia. The treatment was carried out after full surgical excision and there has been no recurrence in 2 years. Two patients died within 4 days, but the remaining 2 patients showed the beginning of recovery as defined above, by the 8th and 11th days respectively. One patient survived 80 days and had regained a normal leucocyte count on the 30th day (Fig. 2). Tumour regression was disappointingly meagre.

*Group II. Mannomustine dihydrochloride* (6 *patients, Cases* 16–21).—The patients in this group also received between 2 and 4 times the usual dose of the drug which was administered in one-half to one-third of the usual time. In spite of this, toxic effects were limited to mild nausea in one case and minimal drowsiness in another. In all cases there was severe leukopenia and thrombocytopenia. The first patient treated with this agent received an infusion of stored autologous bone marrow 24 hr. after the completion of treatment, but in order to allow a longer time for elimination of the drug as well as to permit more complete emptying of the marrow spaces, the remaining patients received their marrow infusions as soon after 4 days as their leucocyte count fell below 1000/cu.mm. Fig. 3 shows a typical response, illustrating well the much slower fall in the leucocyte

TABLE II.—*Autologous Marrow Infusion in Anti-tumour Chemotherapy*

| Case No. | Age and sex | Diagnosis | Chemotherapy: Total dose mg/kg. | Chemotherapy: Duration of course | Tumour regression | Interval between end of chemotherapy and bone marrow infusion | Bone marrow infusion: Number of marrow cells infused ($\times 10^6$/kg.) | Bone marrow infusion: Interval from marrow infusion to beginning of recovery (days) | Survival time: Measured from end chemotherapy (days) | Cause of death |
|---|---|---|---|---|---|---|---|---|---|---|
| *Musine hydrochloride.* Administered by intermittent intravenous injection except for Case 15 who received a limb perfusion | | | | | | | | | | |
| 11 | 56 F. | Carcinoma oesophagus | 1·85 | 15 hours | No | 3 days | 2 | — | 7 | Broncho-pneumonia |
| 12 | 32 M. | Malignant melanoma | 1·30 | 3 days | Slight | 3 „ | 6 | — | 5 | Ditto |
| 13 | 34 F. | Ditto | 1·10 | 11 hours | No | 2·5 hours | 36 | 11 | 15 | Pulmonary metastases |
| 14 | 39 „ | „ | 1·00 | 30 min. | Slight | 6·0 „ | 22 | 8 | 80 | Cerebral metastases |
| 15 | 42 M. | „ | 0·80 | 25 „ | — | 7·0 „ | 130 | — | Alive without recurrence 2 years later | — |
| *Mannomustine hydrochloride.* Administered by intermittent intravenous injection | | | | | | | | | | |
| 16 | 40 F. | Malignant melanoma | 65 | 5 days | Good | 1 day | 9 | — | 8 | Cerebral haemorrhage |
| 17 | 45 M. | Ditto | 47 | 6 „ | Slight | 20 days | 36 | 7 | 84 | Cerebral metastases |
| 18 | 12 „ | Hodgkin's disease | 38 | 2 „ | No | 4 „ | 8 | — | 11 | Myocardial necrosis |
| 19 | 36 „ | Malignant melanoma | 47 | 23 „ | Slight | 11 „ | 103 | 5 | 21 | Cerebral haemorrhage |
| 20 | 64 F. | Ditto | 50 | 26 „ | No | 7 days + 15 days | 50 | 6 | 16 | Broncho-pneumonia |
| 21 | 62 „ | Carcinoma caecum | 20 | 10 „ | „ | 15 „ | 83 | 4 | 78 | Vena Caval thrombosis |
| *Cyclophosphamide.* Administered by intermittent intravenous injection | | | | | | | | | | |
| 22 | 52 F. | Ovarian carcinoma | 40 | 9 days | No | 5 days | 14 | 2 | 126 | No autopsy |
| 23 | 21 M. | Ewing's tumour | 165 | 48 hours | Good | 7 „ | 19 | 7 | 277 | Pulmonary metastases |
| 24 | 67 „ | Carcinoma body of uterus | 67 | 25 „ | Good | 4 „ | 55 | — | 9 | Pulmonary metastases |
| 25 | 42 F. | Pulmonary carcinoma | 67 | 26 „ | No | 6 „ | 4 | 4 | 180 | No autopsy |
| *Phenylalanine mustard.* Administered by continuous intravenous infusions | | | | | | | | | | |
| 26 | 34 F. | Malignant melanoma | 2·30 | 7 days | Good | 50 days | 13 | 12 | 180 | See Case 28 |
| 27 | 39 „ | Ditto | 0·96 | 24 hours | Fair | 6 „ | 41 | — | 7 | Broncho-pneumonia |
| *N-Deacetyl thiocolchicine.* Administered by continuous intravenous infusion | | | | | | | | | | |
| 28 | 34 F. | Malignant melanoma | 1·70 | 24 hours | Good | 3 days | 6 | 4 | 63 | Haemorrhage into cerebral metastases |
| *Triethyleneglycol diglycidyl ether.* Administered as a single intravenous injection | | | | | | | | | | |
| 29 | 34 M. | Malignant melanoma | 300 | 5 min. | No | 12 days + 20 days | 26 and 14 | 13 from 1st | 91 | Pulmonary metastases |

Fig. 2.—Case 14.—Effect of autologous marrow infusion in a patient receiving 1 mg./kg. of mustine hydrochloride.

Fig. 3.—Case 17.—Effect of autologous marrow infusion in a patient receiving 47 mg./kg. of mannomustine hydrochloride.

and platelet counts when comparison is made with mustine hydrochloride, and the rapid recovery after autologous marrow infusion. Three cases died before recovery was complete, two as a result of haemorrhage into necrotic cerebral metastases and one from myocardial necrosis. Tumour regression was greater than with mustine hydrochloride, but was still very disappointing.

*Group III. Cyclophosphamide* (4 *patients, Cases* 22–25)—The total doses of this drug were not remarkable, but the rate at which it was administered was increased up to twentyfold. Side effects were not prominent except in case 23,

FIG. 4.—Case 23.—Effect of autologous marrow infusion in a patient receiving 165 mg./kg. of cyclophosphamide.

who received 9·6 grams in 48 hr. This produced nausea and vomiting and severe limb cramps. Alopecia occurred in all patients who survived longer than 2 weeks. Infusions of stored autologous marrow were again given as soon after 4 days as leukopenia was apparent and very rapid recovery was seen in three patients (Fig. 4) ; Case 24 died as a result of her pulmonary metastases on the 5th post-infusion day. Good tumour regression was seen in 2 cases (Ewings tumour and pulmonary carcinoma), but the remaining 2 showed no response.

*Group IV. Phenylalanine Mustard* (2 *patients, Cases* 26 *and* 27)—This drug was administered by continuous intravenous injection, a technique which seems to result in a distinct increase in clinical effectiveness. Because of this increase in toxicity no precise comparison with divided dosage is possible. Leucopenia developed in both patients and was treated by injection of stored autologous marrow. One patient died as the result of her disease on the following day, but the other patient showed improvement in her leucocyte count following the infusion (Fig. 5). Both patients showed encouraging tumour regression.

*Group V. N-deacetyl thiocolchicine* (1 *patient, Case* 28)—The dose used was about 12-times the normal daily dose, but only one-quarter of the normal dose

FIG. 5.—Case 26.—Effect of autologous marrow infusion in a patient receiving 2·3 mg./kg. of phenylalanine mustard.

for a complete course. Alopecia developed 3 weeks later. Severe leucopenia and thrombocytopenia followed rapidly, and the marrow infusion was given after 3 days ; it was followed by speedy recovery of the blood counts.

*Group VI. Triethyleneglycol Diglycidyl ether* (1 *patient, Case* 29)—Injection of 300 mg./kg. into a running saline infusion produced dizziness and confusion lasting 2 or 3 minutes, and mild nausea which lasted for 2 days. This dose was about 50 per cent greater than that commonly employed. The treatment was followed by leucopenia and thrombocytopenia, taking about 2 weeks to reach minimum values and the bone marrow at this time was grossly hypoplastic, the only remaining cells being lymphocytes, plasma cells and reticulum cells. Stored autologous bone marrow was given at this time and both bone marrow and blood were normal in 3 weeks.

*Discussion*

The precise recovery rates to be expected from the treatment schedules used in these cases is largely a matter of conjecture, since similar doses have not been used without some method of marrow protection. Clearly it would not be justifiable deliberately to obtain controls.

*Group I. Mustine hydrochloride.*—Our personal experience of rapidly administered mustine hydrochloride is limited to single-shot intra-aortic injections of 0·3 mg./kg. These patients experience a moderate leucopenia with recovery beginning on the 12th or 13th day. Block *et al.* (1948) reported that the leucopenia following 0·4 mg./kg. administered in 4 days occurs in the 3rd week following treatment, and usually lasts for rather less than a week. One case, who received 0·86 mg./kg. in 7 days, suffered a leucopenia of 500 cells/cu. mm. and did not recover a normal count until the 12th week. Dameshek *et al.* (1949) using rather smaller doses also noted the maximum leucopenia in the third week with restoration to normal in 5–6 weeks. Bierman *et al.* (1949) described the haematological effects of larger single doses of mustine hydrochloride (0·6 mg./kg.); the maximum leucopenic effect was seen in 11–16 days with recovery in 34–38 days. The rate of recovery in cases 13 and 14 was more rapid than any of these in spite of the much larger doses of mustine being given.

Comparison with the cases reported by Conrad and Crosby (1960) shows that the protection offered is comparable to that provided by the use of orthopaedic tourniquets on the limbs during chemotherapy.

It was surprising to find so little blood count depression after perfusing 0·8 mg./kg. in Case 15. Austin *et al.* (1959) found that the perfusion of this dose with a systemic leak of 2·5 per cent per minute (the measured leak in Case 15) resulted in leucopenia to about 1000/cu. mm. and thrombocytopenia to about 100,000/cu. mm. In two cases studied personally the perfusion of 0·8 mg./kg. resulted in leucopenia with recovery to pre-treatment levels in 3–4 weeks. It seems certain therefore that autologous marrow infusion helped to prevent excessive haemopoietic depression in this case.

*Group II. Mannomustine dihydrochloride.*—In a series of 24 patients receiving normal courses of mannomustine, white cell depression occurred whenever the dose rate exceeded 0·7 mg./kg./day and a cumulative total of 9 mg./kg. was exceeded. Recovery to pretreatment values took 2–3 weeks from the minimum leucocyte count. This finding is in agreement with the observations of others (Sellei and Eckhardt, 1958; Barlow *et al.*, 1959). These authors emphasise the prolonged marrow depressant effect of this drug; bone marrow smears made 6–8 weeks after treatment may still be hypoplastic. For this reason, Sellei recommended that a second course should not be given for 6 months. These comments apply to conventional treatment schedules: in the present series, in spite of the much higher doses, recovery was complete within 7 days of the minimum count in each case—a striking contrast. There was significant correlation between the recovery time and the number of marrow cells infused ($r = -0{\cdot}816$, p lies between 0·05 and 0·02) although the correlation between the recovery time and the drug dosage ($r = 0{\cdot}768$) was not significant (p lies between 0·1 and 0·05). Biopsies showed that the bone marrow was virtually normal in cellularity by the second week after the infusion.

*Group III. Cyclophosphamide.*—Recovery from the effects of this drug is extremely rapid even in the absence of marrow infusion. In a series of 14 patients receiving conventional doses of this drug, 6 patients experienced a leukopenia below 1000 per cu. mm. and in each case the count returned to 2000 and continued to rise within 10 days. Nissen-Mayer and Höst (1960), Stoll and Matar (1961) and Anders and Kemp (1961) also noted the startling difference in speed of recovery when this agent is compared with most other alkylating agents. In view of this rapid recovery, it was considered safe to pair Case 25 with a similar

FIG. 6.—Recovery of leucocyte count in Case 25 who received 67 mg./kg. of cyclophosphamide and autologous bone marrow, contrasted with a control patient who received 67 mg./kg. of cyclosphosphamide without a marrow infusion.

patient who would receive the same dose of cyclophosphamide, but no bone marrow infusion. This patient experienced a rather less severe leukopenia, but recovery occurred at a comparable time and rate (Fig. 6). Case 23 received twice as much cyclophosphamide and recovery occurred 2 days later than in the other cases, but was again at the same rate. Although the infused marrow may have helped this patient, it must be concluded that substantial benefit from marrow infusion after this drug cannot be demonstrated.

*Group IV. Phenylalanine mustard.*—The haematological behaviour of control patients receiving the same dose of alkylating agents as Case 26 has been studied and there was no evidence of an increased rate of peripheral blood recovery when the marrow infusion was given.

*Group V. N-deacetyl thiocolchicine.*—N-deacetyl thiocolchicine has been used without autologous marrow infusion in a dosage of 0·9 mg./kg.: the subsequent depression and recovery of the blood picture was remarkably similar to that observed in Case 28. In view of the considerable difference in drug dosage, it seems possible, but by no means certain, that the marrow infusion accelerated the recovery of this patient.

*Group VI. Triethyleneglycol diglycidyl ether.*—This drug has been given in approximately one half of the dosage used here, to 2 patients who did not receive bone marrow infusion. The leucopenia was less severe, but recovery began at about the same time as it did in Case 29. According to Innman (1962, personal communication) recovery after 300 mg./kg. would be expected to take 28–32 days. It seems probable therefore that the marrow infusion accelerated recovery by a few days.

In the whole series, 13 patients received more than $11 \times 10^6$ marrow cells/kg. in the infusion. The 6 patients who received less than this either failed to recover (Cases 11, 12, 16 and 18) or had received more moderate drug dosage, in which case large marrow infusions would not be expected to be so important (Cases 25 and 28).

### *Homologous Marrow Infusions*

Twenty-three patients were studied.

#### *Results*

Details of the attempted bone marrow homografts are given in Table III. Cases 30–37 were suffering from naturally occurring bone marrow hypoplasias of various types. Two patients received preliminary treatment designed to increase the chance of a successful graft; Case 31 received total body irradiation and case 35, regional irradiation and intravenous mustine hydrochloride. Although there was some haematological improvement in 5 of these 8 cases, in no case was it sufficiently impressive to suggest a successful graft. The polymorph nuclear sex tag was available for study in Cases 35 and 37 and chromosomal analysis for the presence of donor (male) cells in the peripheral blood was carried out in Case 33; none of these cases provided any evidence of a functioning marrow graft.

Cases 38–47 were suffereing from bone marrow hypoplasia due to cancer therapy. Preliminary regional irradiation was given to 2 patients (Cases 38 and 40). Five patients showed some improvement following the marrow infusion, but this was probably not significant in Cases 38 and 44, and in Cases 45–47 the recovery was no more rapid than would be expected in the absence of marrow infusion. Erythrocyte antigen tags were followed in Cases 46 and 47, and the nuclear sex tag in Cases 43 and 44; no case showed any evidence of a successful homograft.

Cases 48–52 were all suffering from malignant disease and were treated particularly energetically, knowing that homologous marrow would be available for infusion. The 2 patients suffering from chronic lymphatic leukaemia were both benefited by the radiotherapy, particularly Case 48. Cases 50 and 51, children with acute leukaemia, were subjected to total body irradiation and homologous marrow infusion. Case 50, who received marrow 30 minutes after

TABLE III.—*Homologous Marrow Infusion*

| Case No. | Age | Sex | Diagnosis | Treatment preceding marrow infusion | Bone marrow infusion | Source of marrow | Survival time | Improvement following marrow infusion |
|---|---|---|---|---|---|---|---|---|
| 30 | 24 | ♀ | Chloramphenicol induced aplastic anaemia | None | $48 \times 10^6$ cells/kg. | * | 47 days | Platelet count rose from 40,000/cu. mm. to 130,000/cu. mm. in 4 days |
| 31 | 54 | ♀ | Aplastic anaemia ? chloramphenicol induced | Total body irradiation 200 rads | $29 \times 10^6$ cells/kg. | Daughter | 8 ,, | Blood counts improving at time of death |
| 32 | 5 | ♀ | Chloramphenicol induced aplastic anaemia | None | $290 \times 10^6$ cells/kg. | Mother | 16 ,, | None |
| 33 | 74 | ♀ | Idiopathic aplastic anaemia | None | $84 \times 10^6$ cells/kg. | Son | 4 months | Improved sternal marrow cellularity |
| 34 | 66 | ♀ | Aplastic anaemia probable toxic aetiology | None | $51 \times 10^6$ cells/kg. | Grandson | 6 weeks | None |
| 35 | 67 | ♂ | Myeloid metaplasia ? toxic aetiology | Irradiation of pelvis and femora and Mustine hydrochloride | $11 \times 10^6$ cells/kg. | Daughter, brother, and nephew | 61 days | Leucocyte count rose from 430/cu. mm. to 2,000/cu. mm. in 40 days |
| 36 | 1 $\frac{7}{12}$ | ♀ | Acute lymphoblastic leukaemia | None | $350 \times 10^6$ cells/kg. | Grandfather | 4 weeks | Transient slight improvement of platelet count |
| 37 | 30 | ♂ | Lymphosarcoma | None | $43 \times 10^6$ cells/kg. | * | 6 months | None |
| 38 | 25 | ♀ | Hodgkin's disease | Radiotherapy, Triethylene thiophosphoramide, Mustine hydrochloride and Chlorambucil | $16 \times 10^6$ cells/kg. | Mother and brother | 3 days | Leucocyte count rose from 4500/cu. mm. to 6500/cu. mm. in 3 days |
| 39 | 11 | ♂ | Lymphosarcoma and leukaemia | Radiotherapy, Amethopterin and 6-Mercaptopurine | $9 \times 10^6$ cells/kg. | Mother | 8 days | None |
| 40 | 14 | ♀ | Neuroblastoma | Radiotherapy | $30 \times 10^6$ cells/kg. | Father and mother | 15 min. | None |
| 41 | 38 | ♀ | Hodgkin's disease | Radiotherapy, Mannomustine hydrochloride and Chlorambucil | $32 \times 10^6$ cells/kg. | * | Lost to follow-up at 1 month | None |
| 42 | 59 | ♂ | Anaplastic sarcoma of lymphoid tissue | Radiotherapy and Mannomustine hydrochloride | $15 \times 10^6$ cells/kg. | * | 11 days | None |
| 43 | 64 | ♂ | Lymphosarcoma | Radiotherapy, Chlorambucil and 6-Mercaptopurine | $16 \times 10^6$ cells/kg. | * | 6 days | None |
| 44*a* | 30 | ♂ | Acute monocytic leukaemia | 6–Mercaptopurine | $11 \times 10^6$ cells/kg. | * | 4 months | None |
| 44*b* | 30 | ♂ | Ditto | ,, | $48 \times 10^6$ cells/kg. | Wife | 2 months | The platelet count rose from 30,000/cu. mm. to a maximum of 100,000/cu. mm. in 23 days |
| 45 | 49 | ♀ | Carcinoma of stomach | Mustine hydrochloride | $23 \times 10^6$ cells/kg. | * | 11 days | Leucocyte count rose from 300/cu. mm. to 13,000/cu. mm. in 9 days |
| 46 | 48 | ♀ | Ovarian carcinoma | Radiotherapy and Cyclophosphamide | $41 \times 10^6$ cells/kg. | Sister | 3 months | Leucocyte count rose from 1/cu. mm. to 4000/cu. mm. in 14 days |

TABLE III—*cont.*

| Case No. | Age | Sex | Diagnosis | Treatment preceding marrow infusion | Bone marrow infusion | Source of marrow | Survival time | Improvement following marrow infusion |
|---|---|---|---|---|---|---|---|---|
| 47 | 45 | ♀ | Carcinoma of breast | Radiotherapy and Triethylene thiophosphoramide | $174 \times 10^6$ cells/kg. | Son | 10 ,, | Leucocyte count rose from 400/cu. mm. to 4000/cu. mm. in 30 days |
| 48*a* | 66 | ♀ | Chronic lymphatic leukaemia | Pelvic irradiation 2500 rads in 20 days | $17 \times 10^6$ cells/kg. | Sons and daughters | 16 ,, | None |
| 48*b* | 67 | ♀ | Ditto | Lower half body irradiation, 520 rads in one treatment | $22 \times 10^6$ cells/kg. | * | 12 days | None |
| 49 | 56 | ♂ | Ditto | Total body irradiation in two half-body fractions of 800 rads | $144 \times 10^6$ cells/kg. | * | 3 months | None |
| 50 | 8 | ♂ | Lymphosarcoma and leukaemia | Total body irradiation, 500 rads | $40 \times 10^6$ cells/kg. | Mother | 23 days | None |
| 51 | 6 | ♀ | Acute lymphoblastic leukaemia | Total body irradiation, 1160 rads in 8 half-body fractions | $67 \times 10^6$ cells/kg. | Father | 14 days | Both leucopenia and thrombocytopenia improving at time of death |
| 52 | 12 | ♂ | Hodgkin's disease | Mustine hydrochloride 0·6 mg./kg. in 3 days | $79 \times 10^6$ cells/kg. | * | 3 months | Leucocyte count recovered from a minimum of 200/cu. mm. to 3000/cu. mm. in 28 days |

* Ribs removed at thoracotomy and marrow stored at − 79° C.

the irradiation, failed to show any recovery by the 14th day. He was then given a foetal liver infusion but still failed to respond and died with bone marrow aplasia on the 23rd day. Active tumour tissue was in evidence at the autopsy. Case 51 was slightly more successful.

This child was treated at the beginning of the second relapse : two remissions had been obtained previously with amethopterin and 6-mercaptopurine respectively, Prednisone having been given throughout. Whole body irradiation was administered with the 2 MeV X-ray generator in daily half-body fractions, each half receiving 4 treatments at 2-day intervals. The total centre dose was 1160 r. The equivalent single exposure was clearly less than this, and an estimate based on the studies of Elkind and Sutton (1959) indicated an equivalent of about 860 r.

Bone marrow was administered 3 days after the last radiation fraction to allow good time for aplasia to develop. Serial blood counts (Fig. 7) indicated the beginning of recovery at about 7 days after infusion. At the time of death, 14 days from the infusion, the platelet count had reached 153,000 per cu. mm. The cause of death was an overwhelming monilial infection of the oesophagus and stomach, and *E. coli* septicaemia.

The speed of the recovery of the blood count of Case 52 after treatment with mustine hydrochloride was probably no more rapid than it would have been without the marrow infusion. The female neutrophil tag was applicable to Cases 49, 50 and 52, and in Case 49 female polymorphs were, indeed, detected between the 42nd and 45th day after the infusion. Red cell antigen studies were also applied to this case, but without any evidence of a functional graft.

*Discussion*

Haematological improvement was observed in 12 of these 23 cases, but only in Case 51 was it sufficiently rapid to suggest a successful homograft. Studies of blood group antigens, polymorphonuclear sex and leucocyte chromosome configuration provide a much more satisfactory test for the " take " of bone marrow grafts, but except for the brief appearance of female polymorphs in one case, these tests also failed to detect successful marrow homografts. In fact,

FIG. 7.—Case 51.—Effect of homologous marrow transfusion in a case of acute leukaemia who received 1160 r (fractionated) of whole-body irradiation.

only 4 cases received infusions which satisfied the minimum requirement of $130 \times 10^6$ cells/kg. : one of these patients—Case 49—was the only case to provide evidence of a successful homograft.

We must conclude that attempts to produce marrow homografts in these patients probably succeeded temporarily in one case and were perhaps successful in another. In neither case was the procedure beneficial to the patient.

*The Place of Marrow Infusion Treatment in Clinical Medicine*

The place of autologous marrow infusion in clinical radiotherapy rests on many factors besides the purely haematological considerations which have been our principal concern thus far, and it seems that the usefulness of whole body and whole trunk irradiation in association with autologous marrow infusion is severely restricted : rapidly growing tumours recur so soon that useful remissions are not obtained. It is possible that the treatment may be indicated in widely disseminated, moderately rapidly growing lymphoid tumours and seminomata. The use of autologous marrow infusions in the treatment of marrow aplasia caused by regional irradiation was intended to increase the patient's total bone marrow reserve ; this could be important should subsequent courses of treatment be needed.

Before considering the place of autologous marrow infusion in association with cancer chemotherapy, it is important to emphasise that there are very significant differences between the effects of alkylating agents and those of ionising radiation. One such difference is that the margin of dosage separating " marrow death " from intestinal death or other lethal effects is very slight ; this is referred to further below. Another difference is that bone marrow regeneration is usually much more rapid after chemotherapy. In the case of cyclophosphamide the speed of natural recovery is so rapid that marrow infusion has no detectable effect even at very high doses. After mustine hydrochloride the recovery is somewhat slower and an acceleration of regeneration is possible after large doses, although the more rapid recovery after low doses renders marrow infusions ineffective (Smiley *et al.*, 1961). However it is only after drugs like mannomustine which produce prolonged leucopenia that obvious benefit comes from autologous marrow infusion. The principal indication for autologous marrow infusion in cancer chemotherapy is provided by the occurrence of severe leucopenia and thrombocytopenia after treatment with an agent known to have a prolonged effect.

It is usually accepted that chemotherapy should be controlled in such a way as to avoid severe bone marrow depression. When autologous marrow infusion is introduced as a planned procedure, this policy is abandoned and severe bone marrow depression is deliberately induced in the hope that greater tumour regression will occur. Such a procedure is indeed precarious, for one is treating patients in the narrow zone of dosage which separates severe bone marrow depression from lethal systemic toxicity. And this is not the only problem. The necessity to wait for the majority of the drug to be eliminated means that the course of treatment must be short, otherwise the patient is exposed to the dangers of leucopenia and thrombocytopenia at a time when the marrow cannot be infused. This has introduced an additional severe limitation to the technique, for it is now becoming increasingly clear that the most effective tumour response is often obtained with protracted therapy. Certainly, none of our patients showed a degree of tumour regression greater than any we have seen in similar patients receiving prolonged low dosage chemotherapy. As a deliberate part of a planned treatment, it is indicated only if the drug which is used is one which produces prolonged haemopoietic depression. In addition, however, we have considered it wise to use the technique as an " insurance policy " when new drugs and treatment schedules are being explored and unintentional bone marrow depression is an ever-attendent risk. It is established that autologous marrow infusion can produce acceleration of marrow regeneration and that this may indeed be very considerable in special circumstances. Lajtha (1960) and others have postulated that conservative measures, including platelet transfusions, antibiotics and barrier nursing, can help these patients. The available animal experimental evidence as well as considerable clinical experience show that such measures do not posses the potency of autologous marrow infusions in these cases.

Homologous marrow infusions have been used in the treatment of aplasic anaemia, bone marrow hypoplasia induced by cancer chemotherapy and leukaemia after whole body irradiation. We have been wholly unsuccessful in benefiting any of these patients, but at the present time the antigenic barriers which prevent acceptance of homografts are largely unknown and further experience may alter the position. The work of Hewitt and Wilson (1959, 1960) has shown conclusively that there is no theoretical justification for the treatment of acute leukaemia by large doses of whole body irradiation and homologous marrow infusion, while the reports of Mathé and his collaborators (Mathé *et al.*, 1960) have emphasised that there is a danger of producing secondary disease in these patients. The lack of success obtained so far merits the application of this technique only in particularly desperate circumstances (Bielby *et al.*, 1960 ; Mathé *et al.*, 1959).

### SUMMARY AND CONCLUSIONS

1. Eight patients have been treated with total thoracic irradiation to 2500–3058 r and have subsequently received intravenous infusions of stored autologous bone marrow. Sternal bone marrow recovery has been somewhat increased by this procedure.

2. One patient receiving trunk irradiation to a dose of 560 r recovered rapidly after autologous marrow infusion.

3. One patient receiving total body irradiation to a centre dose of 300–350 r showed rapid recovery after autologous marrow infusion.

4. In a study of nineteen patients treated with chemotherapeutic agents it was found that in those who had received large doses of mustine hydrochloride and mannomustine dihydrochloride, the rapidity of haematological recovery was increased, and a less marked benefit was noted in patients treated with N-deacetylthiocolchicine and triethyleneglycol diglycidyl ether. In those cases treated with cyclophosphamide and phenylalanine mustard, no benefit from autologous marrow infusion was detected with the doses employed.

5. Twenty-three patients received homologous marrow infusions. Twelve showed subsequent improvement of the blood picture, but this was not due to successful bone marrow homografts. Successful, albeit transient, grafting occurred in one case. A second patient probably had a " take ", but she died before proof could be obtained.

The authors wish to acknowledge the receipt of most generous grants from the British Empire Cancer Campaign, including a full time research grant for one of us (D. E. P.).

We would also like to acknowledge the very great assistance afforded by the Medical and Surgical Staff of Westminster Hospital, and to thank the Medical Committee for permission to publish details of patients treated in the Hospital. We are especially grateful to Mr. G. Westbury for permission to include details of patients treated by regional isolation perfusion, to Dr. W. J. D. Flemming of the Royal Free Hospital for details of Case 21, and to Dr. G. T. Stewart of Queen Mary's Hospital for Children for details of Case 36.

Dr. K. L. G. Goldsmith, of the Blood Group Reference Laboratory, The Lister Institute, was kind enough to undertake blood group analyses in several cases, and Dr. N. H. Kemp of St. George's Hospital, Department of Haematology, carried out the chromosome analyses. Dr. H. E. M. Kay and Dr. M. Constandoulakis of the Royal Marsden Hospital provided the foetal liver cell suspension for Case 50.

We are also especially grateful to Dr. P. Hansell and the staff of the Department of Photography and Illustration, Westminster Medical School, for their kind assistance.

### REFERENCES

ANDERS, C. J. AND KEMP, N. H.—(1961) *Brit. med. J.*, ii, 1516.

ANDREWS, G. A., SITTERSON, B. W., KRETCHMAR, A. L. AND BRUCER, M.—(1959) *Hlth Phys.*, **2**, 134.

AUSTIN, W. G., MONACO, A. P., RICHARDSON, G. S., BAKER, W. H., SHAW, R. S. AND RAKER, J. W.—(1959) *New Engl. J. Med.*, **261**, 1037.

BARLOW, A. M., LEEMING, J. T. AND WILKINSON, J. F.—(1959) *Brit. Med. J.*, ii, 208.

BARNES, D. W. H. AND LOUTIT, J. F.—(1954) Radiobiology Symposium. Ed. Bacq, Z. M. and Alexander, P. London (Butterworths Scientific Publishers), 1955, p. 134.—(1955) *J. nat. Cancer Inst.*, **15**, 901.

BEILBY, J. O. W., CADE, I. S., JELLIFFE, A. M., PARKIN, D. M. AND STEWART, J. W.—(1960) *Brit. med. J.*, i, 96.

BIERMAN, H. R., SHIMKIN, M. B., METTIER, S. R., WEAVER, J., BERRY, W. C. AND WISE, S. P.—(1949) *Calif. Med.*, **71**, 117.

BLOCK, M., SPURR, C. L., JACOBSON, L. O. AND SMITH, T. A.—(1948) *Amer. J. clin. Path.*, **18**, 671.

CONRAD, M. E. AND CROSBY, W. H.—(1960) *Blood*, **16**, 1089.

DAMESHEK, W., WEISEFUSE, L. AND STEIN, T.—(1949) *Ibid.*, **4**, 338.

DENSTAD, T.—(1943) *Acta radiol., Stockh.*, Suppl. No. 52.

DUNJIC, A. AND MAISIN, J.—(1960) *Rev. franç. Ét. clin. Biol.*, **5**, 268.

ELKIND, M. M. AND SUTTON, H.—(1959) *Nature, Lond.*, **184**, 1293.

HEWITT, H. B. AND WILSON, C. W.—(1959) *Brit. J. Cancer*, **8**, 69.—(1960) *Ibid.*, **14**, 186.

HUMBLE, J. G. AND NEWTON, K. A.—(1958) *Lancet*, i, 142.

JACOBSON, L. O., MARKS, E. K., GASTON, E. O., ROBSON, M. AND ZIRKLE, R. E.—(1949*a*) *Proc. Soc. exp. Biol., N.Y.*, **70**, 740.

*Idem*, MARKS, E. K., ROBSON, M. J., GASTON, E. O. AND ZIRKLE, R. E.—(1949*b*) *J. Lab. clin. Med.*, **34**, 1538.

*Idem*, SIMMONS, E. L., MARKS, E. K., ROBSON, M. J., BETHARD, W. F. AND GASTON, E. O.—(1950) *Ibid.*, **35**, 746.

JAMMET, H., MATHÉ, G., PENDIC, B., DUPLAN, J. F., MAUPIN, B., LATARJET, R., KALIC, D., SCHWARZENBERG, L., DJUKIC, Z. AND VIGNE, J.—(1959) *Rev. franç. Ét. clin. Biol.*, **4**, 210.

KAY, H. E. M. AND KOLLER, P. C.—(1960) *CA* (*N.Y.*), **10**, 33.

LAJTHA, L. G.—(1960) *Brit. J. Radiol.*, **33**, 588.

LORENZ, E., CONGDON, C. C. AND UPHOFF, D.—(1952) *Radiology*, **58**, 863.

*Idem*, UPHOFF, D., REID, T. R. AND SHELTON, E. J.—(1951) *J. nat. Cancer Inst.*, **12**, 197.

MATHÉ, G., BERNARD, J., DE VRIES, M. J., SCHWARZENBERG, L., LARRIEU, M. J., LALANNE, C. M., DUTRIEUX, A., AMIEL, J. L. AND SURMONT, J.—(1960) *Rev. Hemat.*, **15**, 115.

*Idem*, JAMMET, H., PENDIC, B., SCHWARZENBERG, L., DUPLAN, J. F., MAUPIN, B., LATARJET, R., LARRIEU, M. J., KALIC, D. AND DJUKIC, Z.—(1959) *Rev. franç. Ét. clin. Biol.*, **4**, 226.

NISSEN-MAYER, R. AND HÖST, H.—(1960) *Chemother. Rep.*, **9**, 51.

PEGG, D. E.—(1960) *Lancet*, i, 682.—(1962) *Brit. J. Cancer*, **16**, 400.

*Idem* AND KEMP, N. H.—(1960) *Lancet*, ii, 1426.
*Idem* AND TROTMAN, R. E.—(1959) *Brit. J. clin. Path.*, **12**, 477.
POLGE, C. AND LOVELOCK, J. E.—(1952) *Vet. Rec.*, **64**, 396.
SELLEI, C. AND ECKHARDT, S.—(1958) *Ann. N.Y. Acad. Sci.*, **68**, 1164.
SMILEY, R. K., MARTIN-VILLAR, J. AND BELANGER, L. F.—(1961) *Canad. med. Ass. J.*, **84**, 1230.
STEWART, J. W.—(1958) Paper read to the Congress of the International Society of Haematology, Rome, 1958.
*Idem*, AND DISCHE, S.—(1956) *Lancet*, ii, 1063.
STOLL, B. A. AND MATAR, J. H.—(1961) *Brit. med. J.*, ii, 283.
SYKES, M. P., CHU, F. C. H. AND WILKERSON, W. G.—(1960) *Radiology*, **75**, 919.
*Idem*, WILKERSON, W. G. AND CHU, F. C. H.—(1959) Paper read to the International Radiology Congress, Munich, 1959.
TALBOT, T. R. AND ELSON, L. A.—(1958) *Nature, Lond.*, **181**, 684.
TRAN BA LOC, MATHÉ, G. AND BERNARD, J.—(1958) *Rev. franç. Ét. clin. Biol.*, **3**, 461.
TUBIANA, M., BERNARD, C. I. AND LALANNE, C.—(1959) *Acta radiol., Stockh.*, **52**, 321.
WESTON, J. K., MAXWELL, R. E., LEE, M., FENZEL, J. AND FISCKEN, R. A.—(1957) *Fed. Proc.*, **16**, 377.


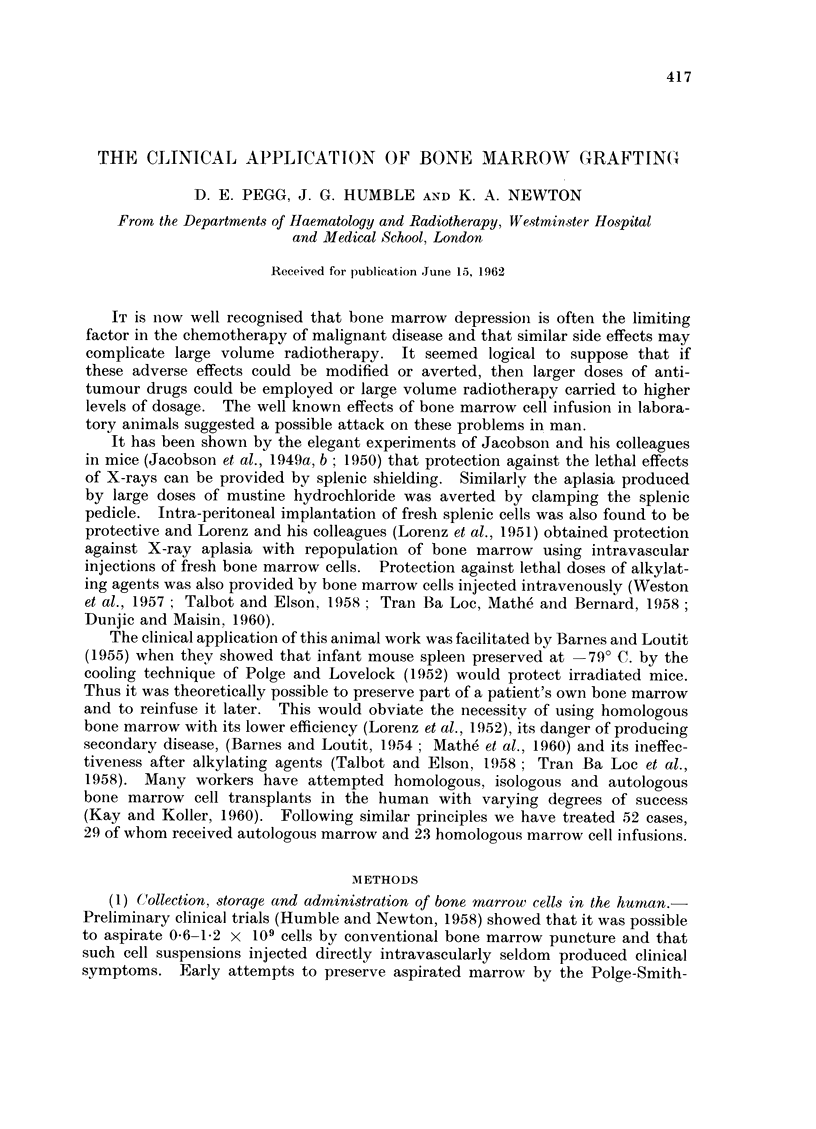

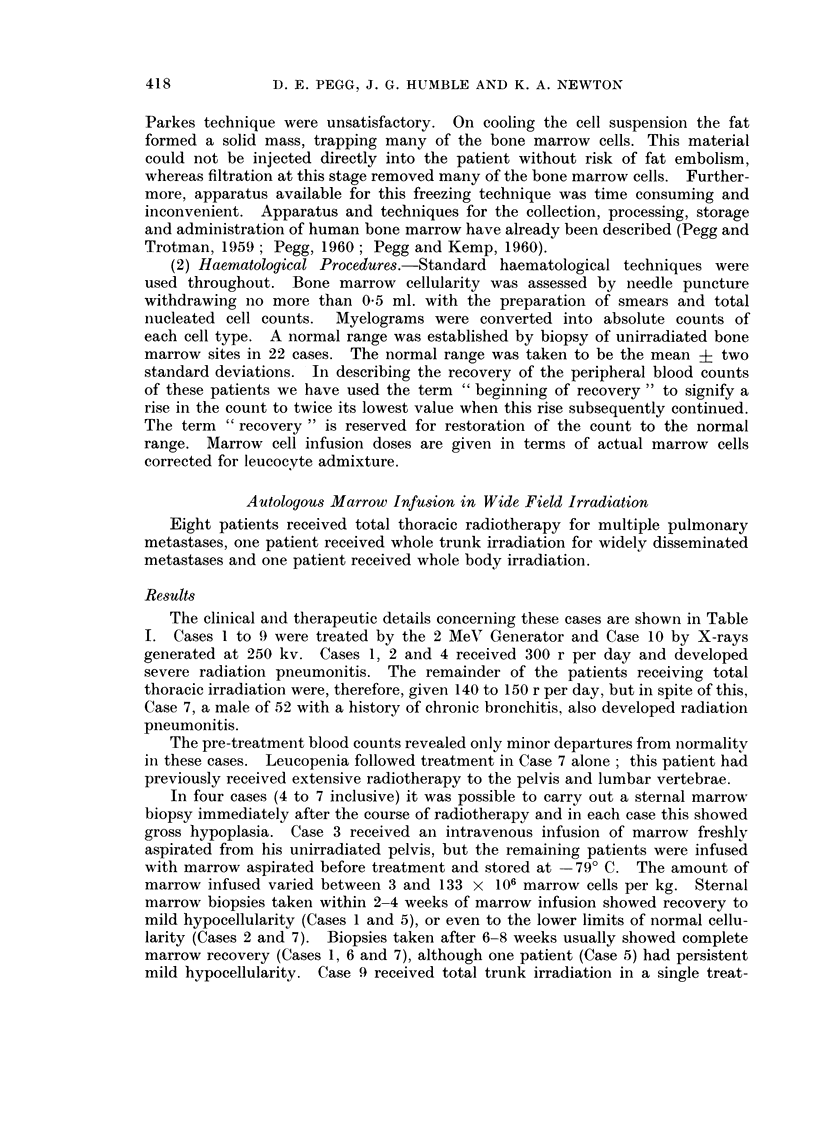

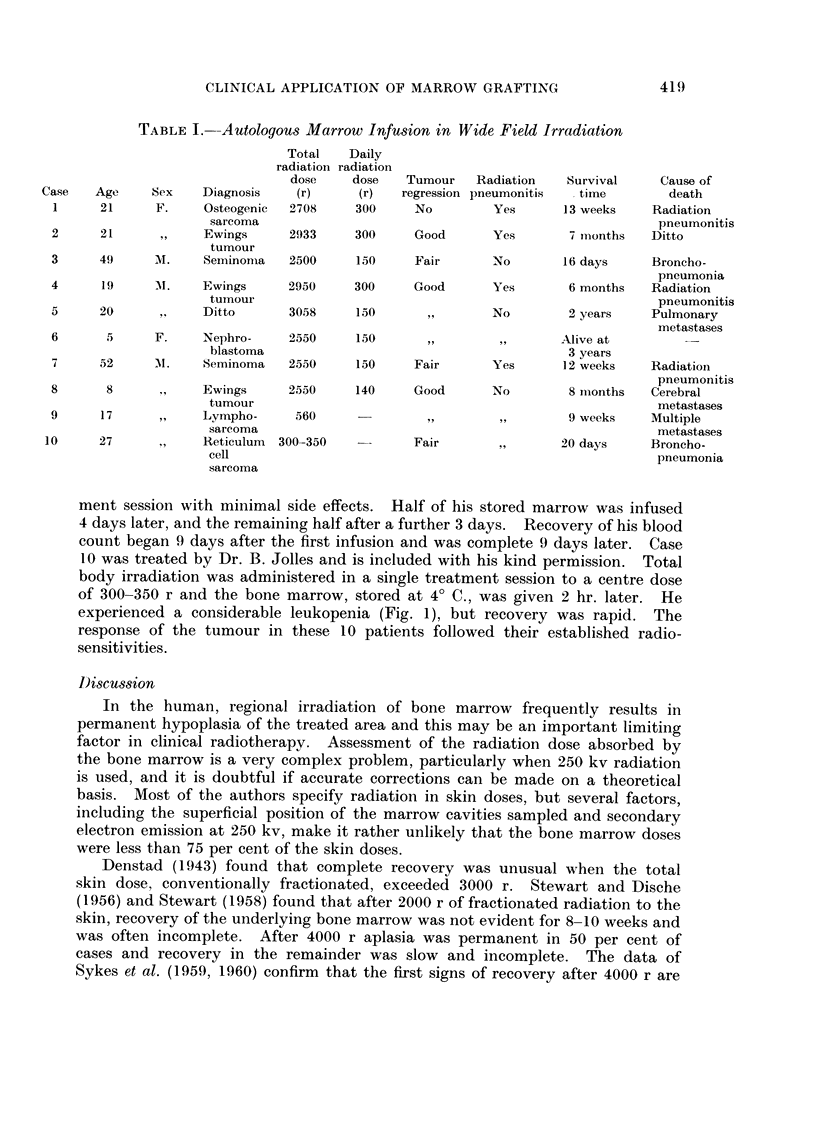

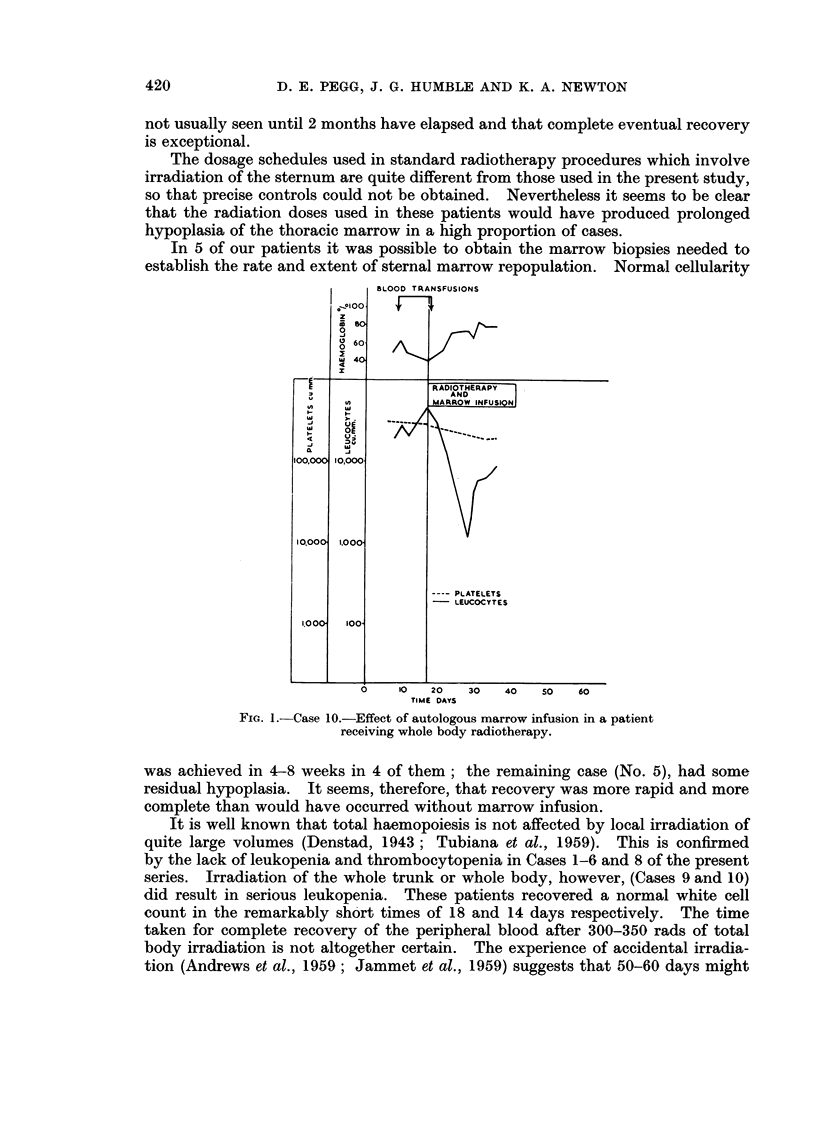

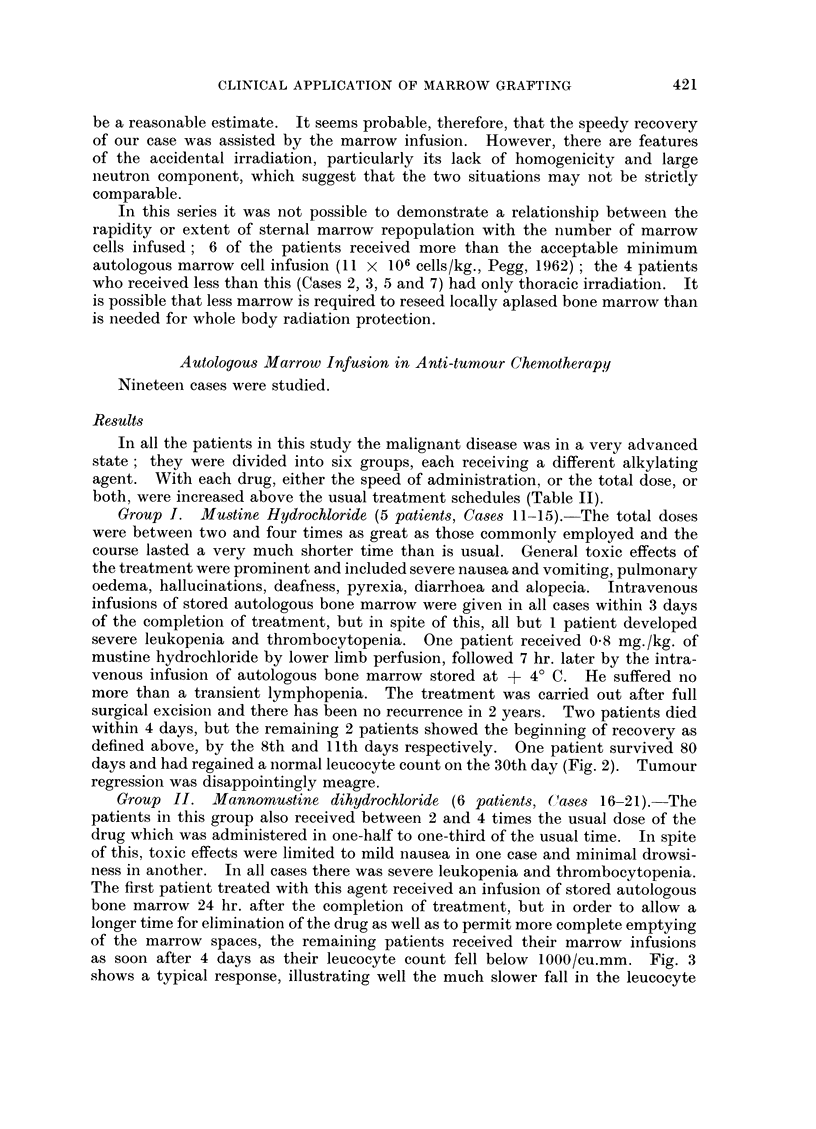

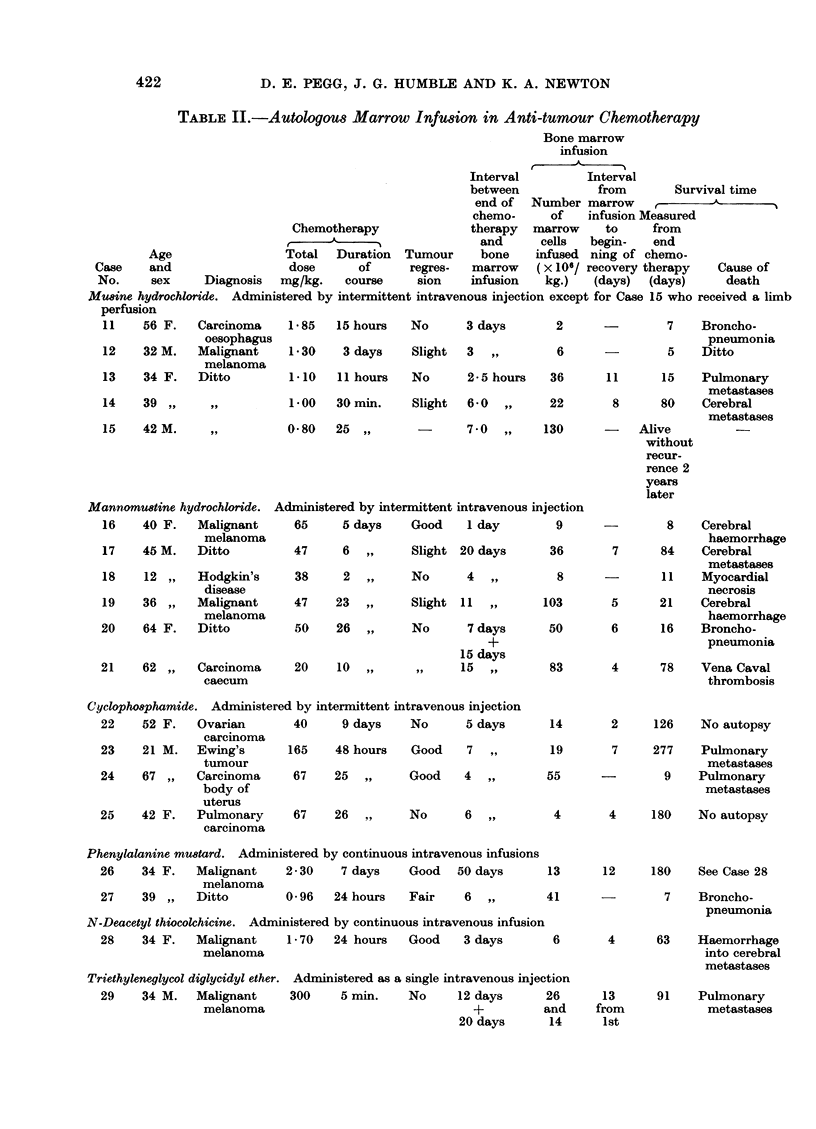

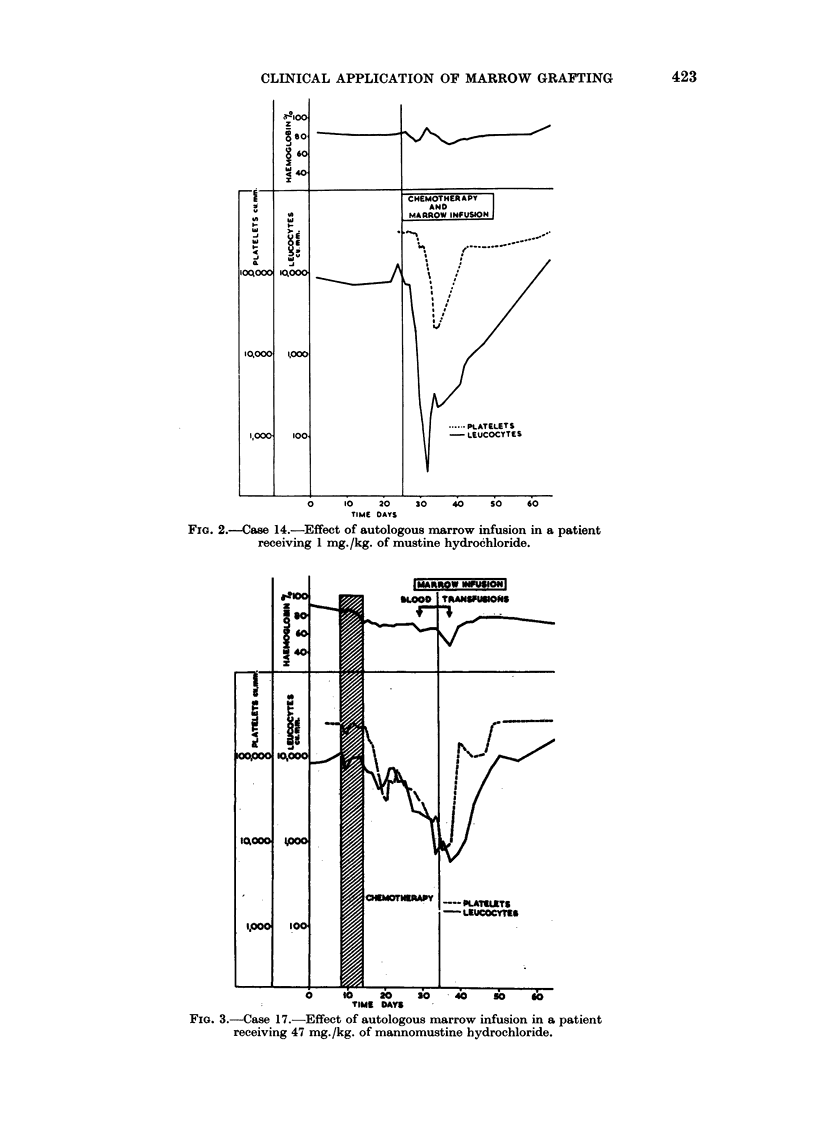

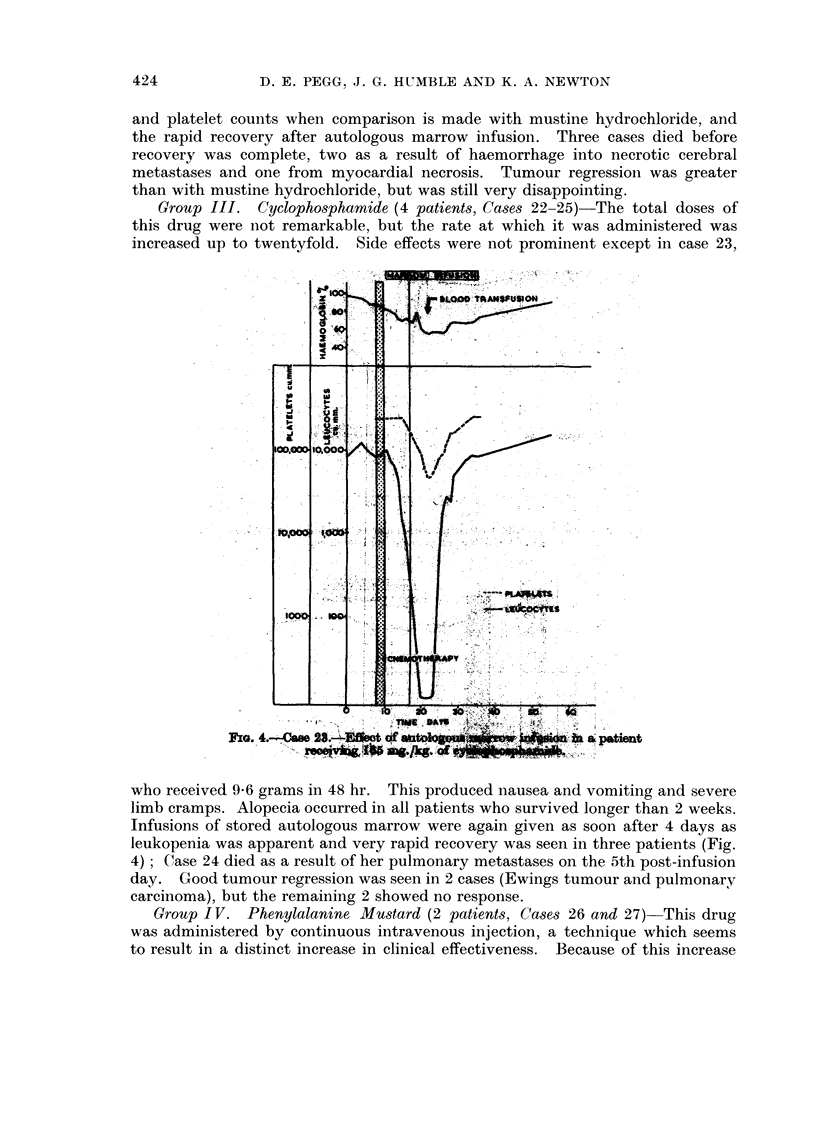

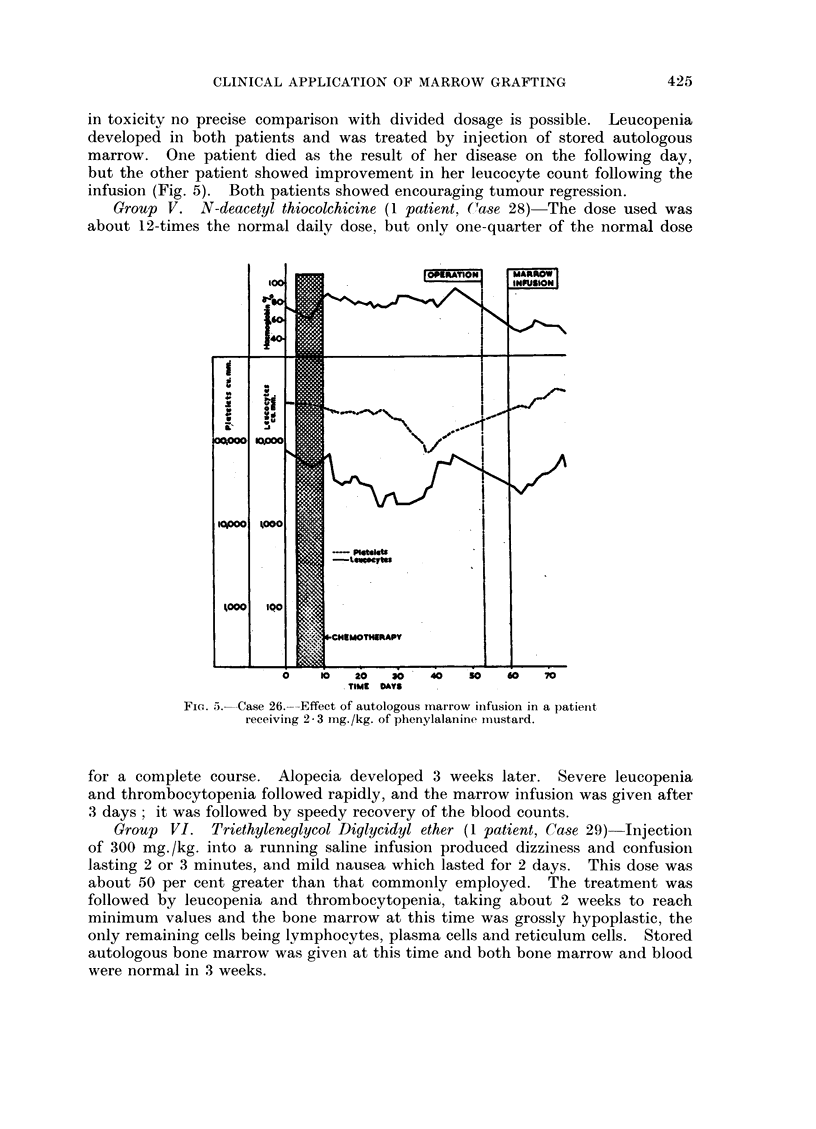

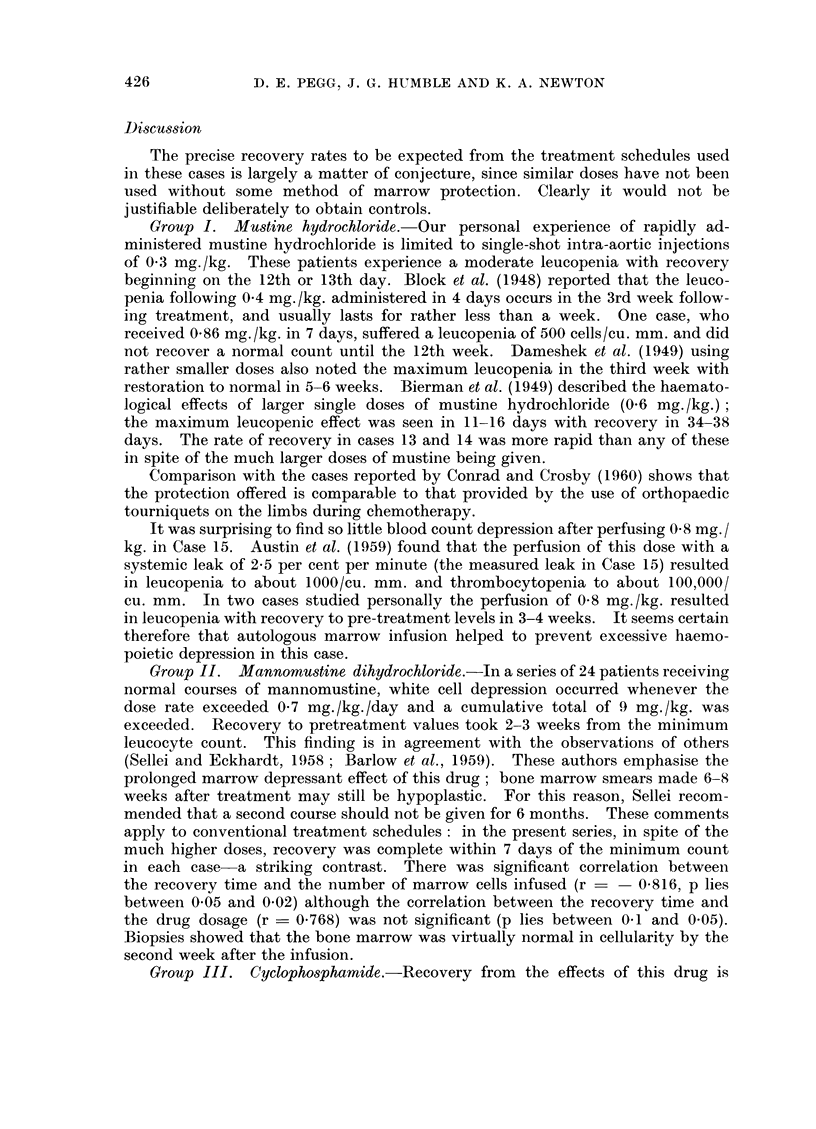

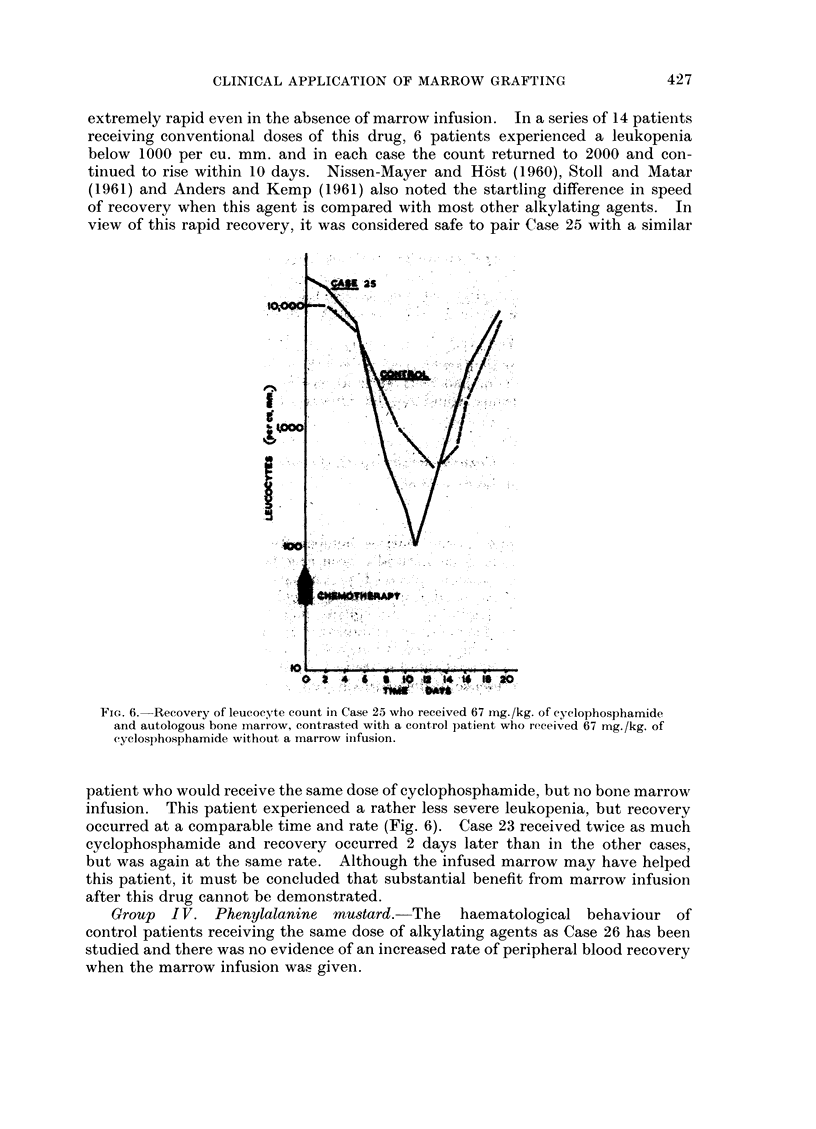

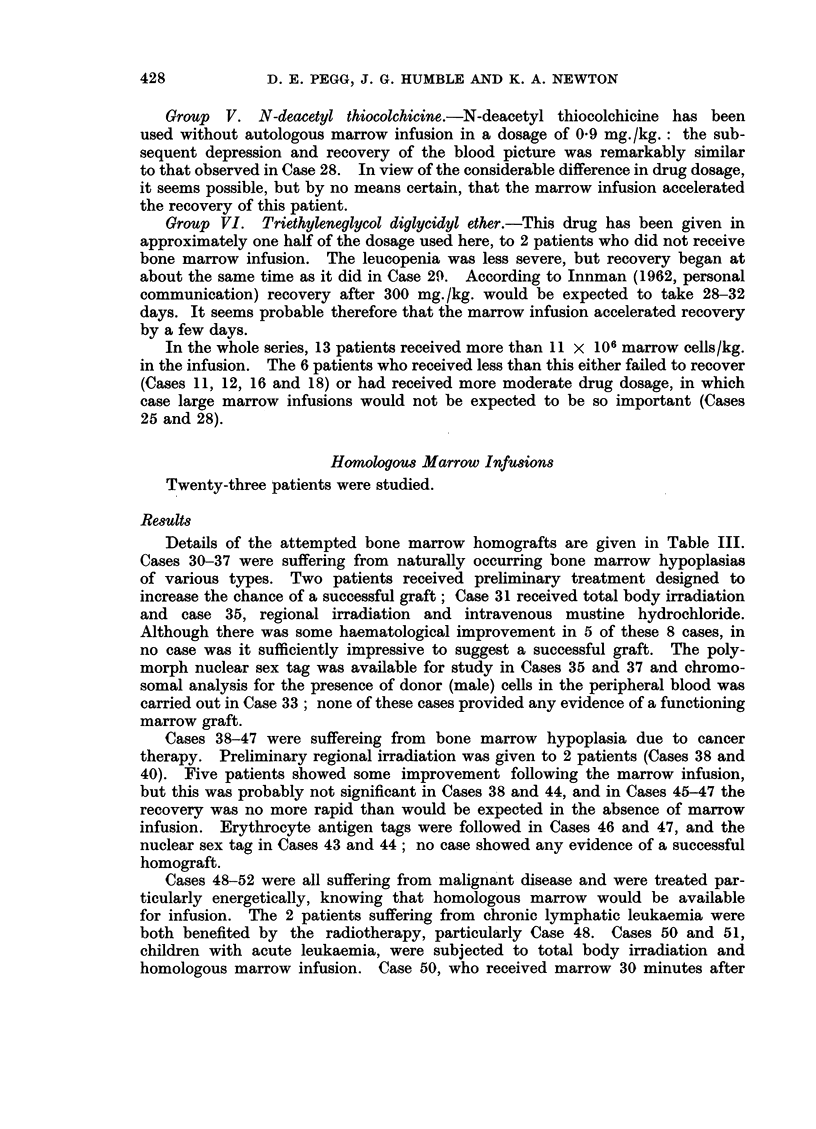

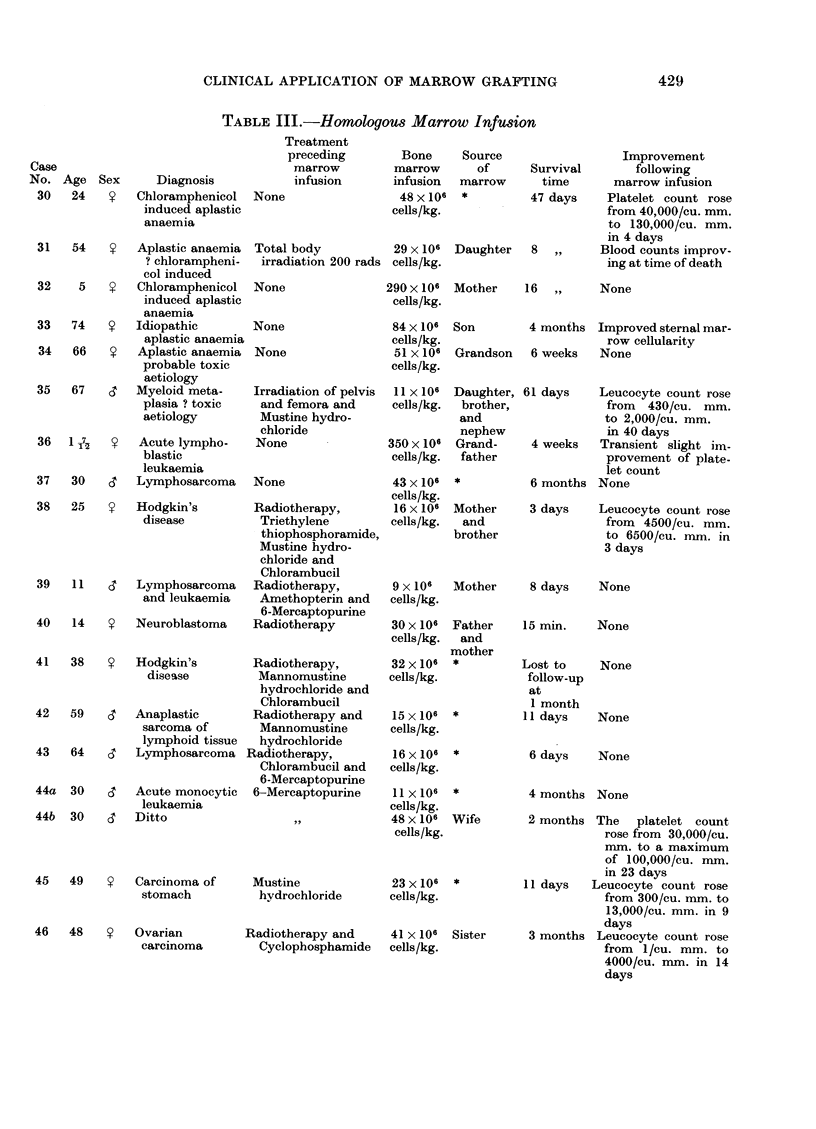

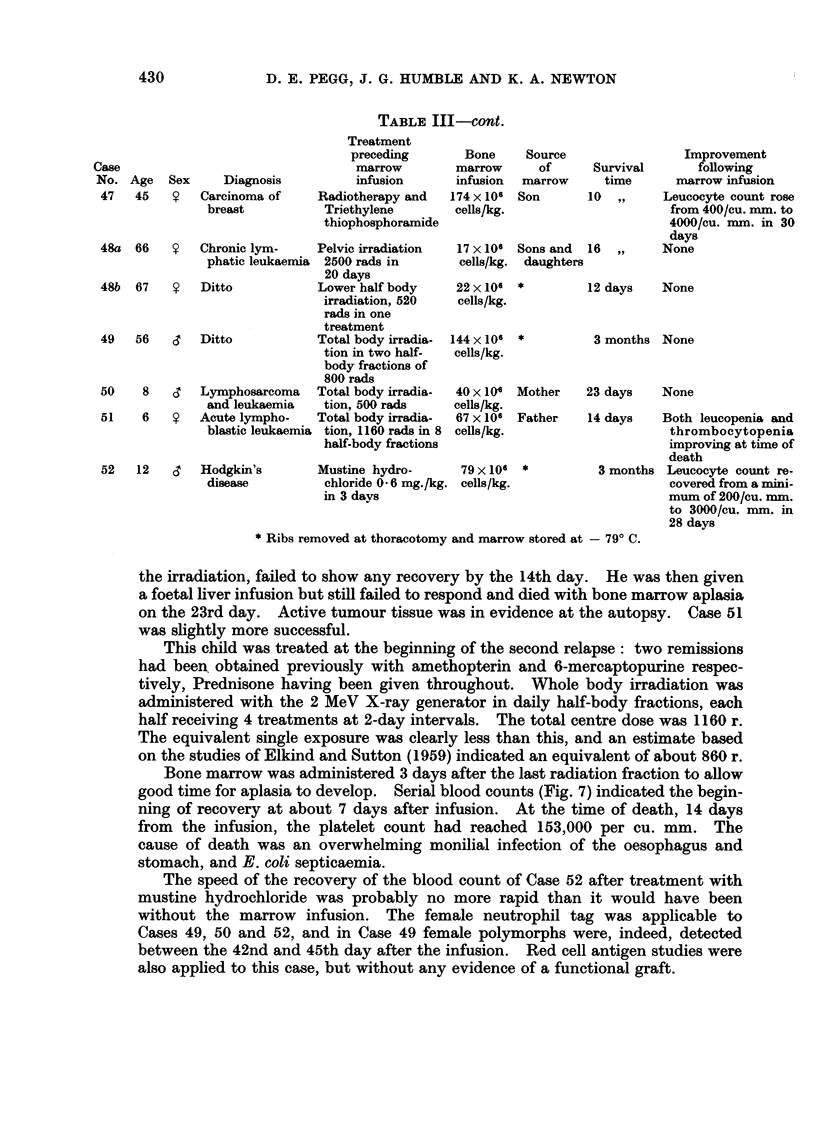

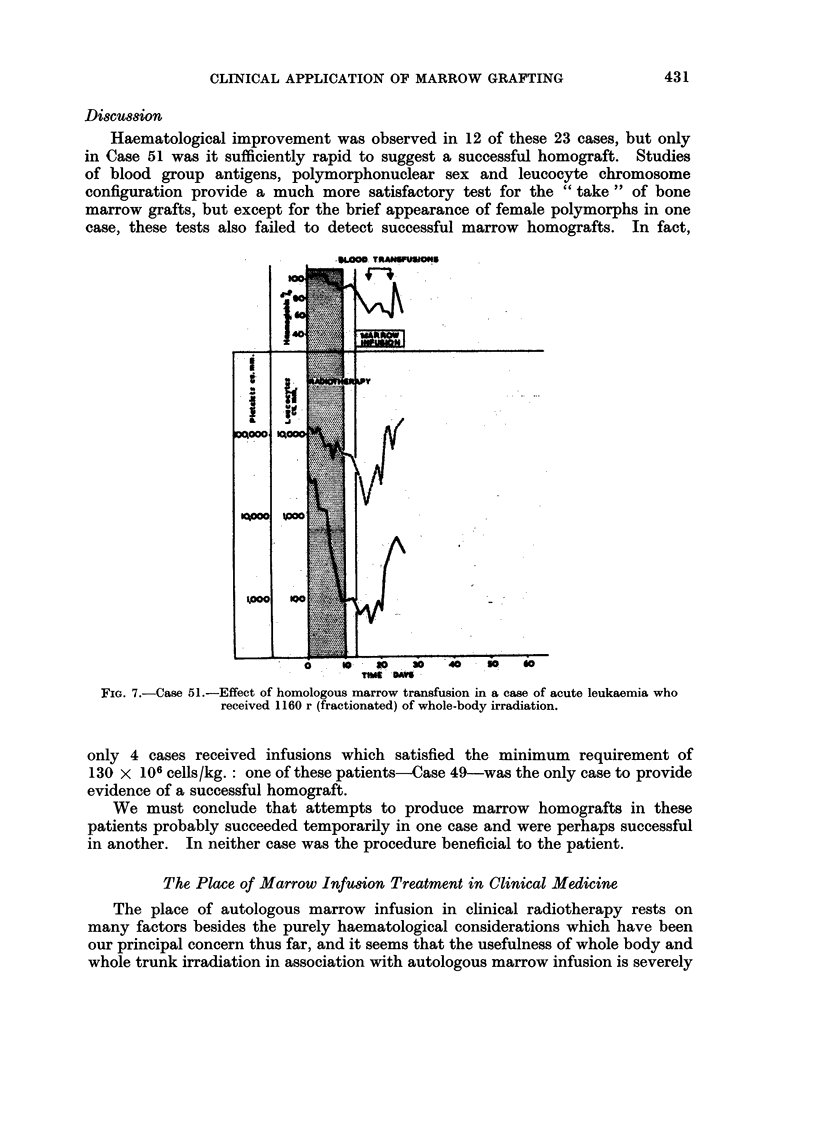

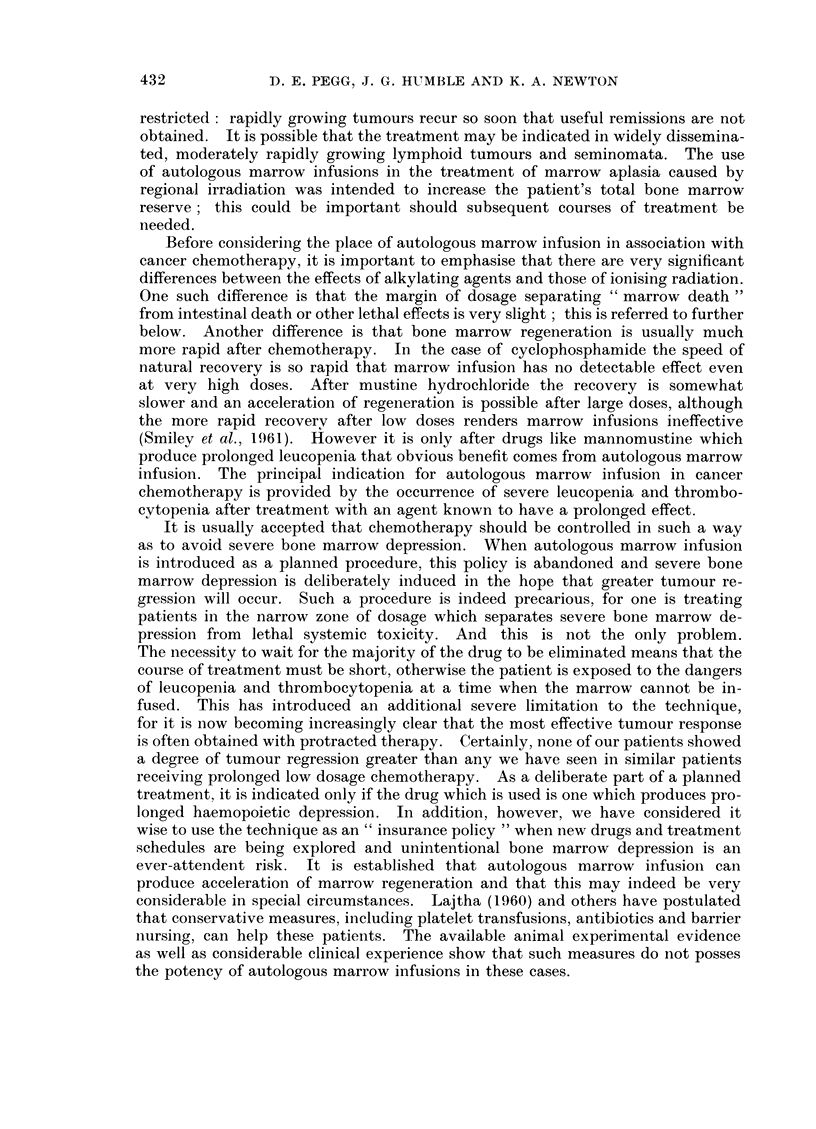

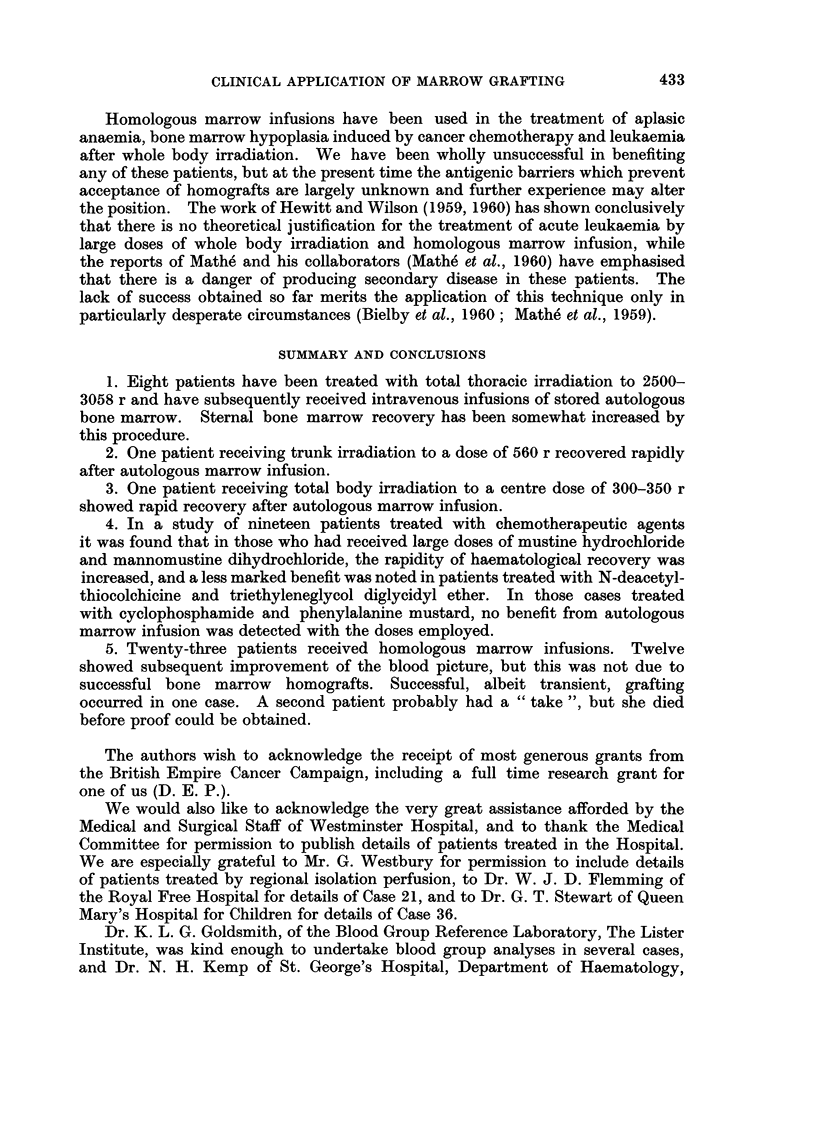

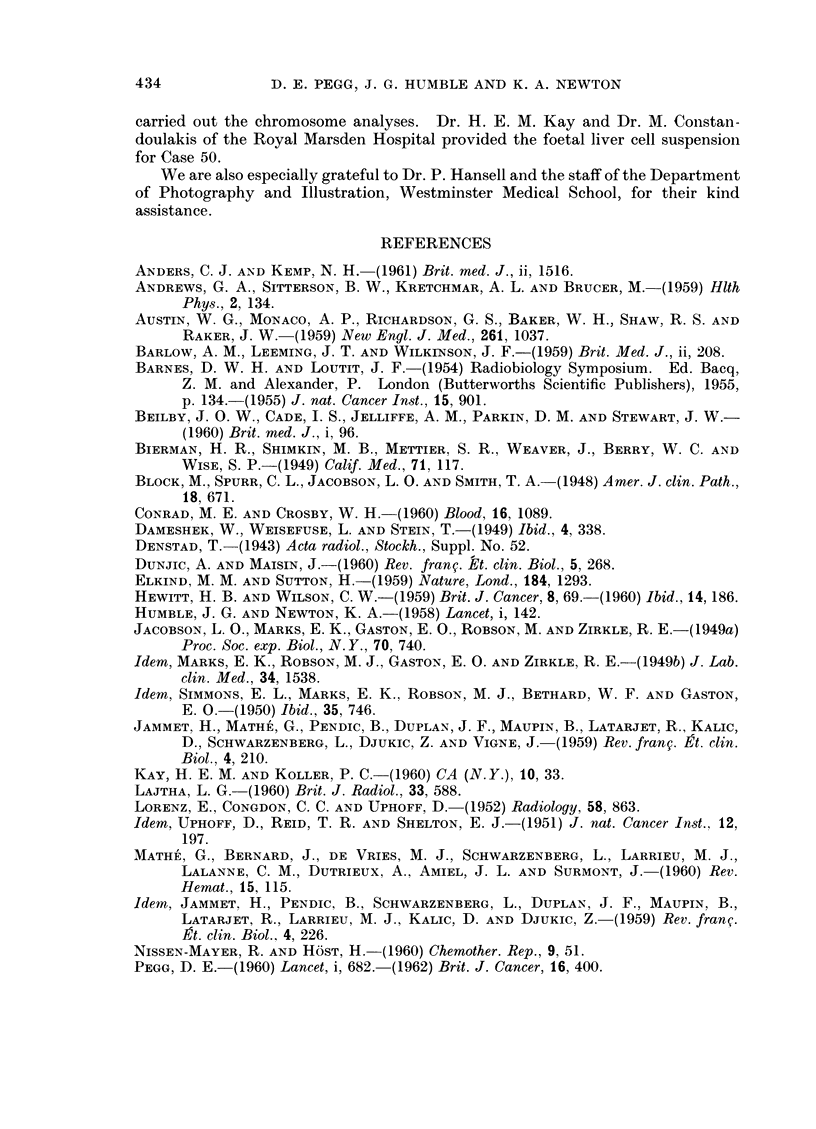

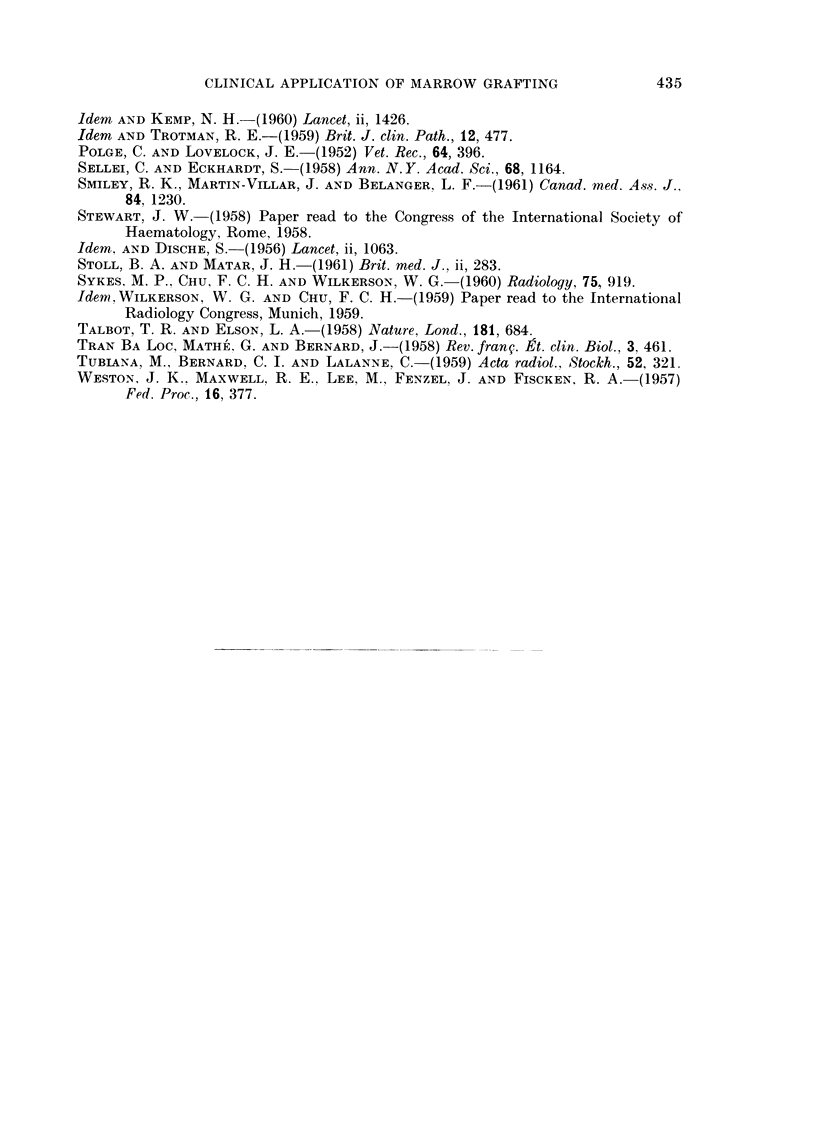


## References

[OCR_01511] ANDERS C. J., KEMP N. H. (1961). Cyclophosphamide in treatment of disseminated malignant disease.. Br Med J.

[OCR_01513] ANDREWS G. A., SITTERSON B. W., KRETCHMAR A. L., BRUCER M. (1959). Accidental radiation excursion at the Oak Ridge Y-12 plant-IV. Preliminary report on clinical and laboratory effects in the irradiated employees.. Health Phys.

[OCR_01517] AUSTEN W. G., MONACO A. P., RICHARDSON G. S., BAKER W. H., SHAW R. S., RAKER J. W. (1959). Treatment of malignant pelvic tumors by extracorporeal perfusion with chemotherapeutic agents.. N Engl J Med.

[OCR_01521] BARLOW A. M., LEEMING J. T., WILKINSON J. F. (1959). Mannomustine in treatment of leukaemias, polycythaemia, and malignant disorders.. Br Med J.

[OCR_01532] BIERMAN H. R., SHIMKIN M. B. (1949). Methyl-bis (beta-chloroethyl)amine in large doses in the treatment of neoplastic diseases.. Calif Med.

[OCR_01540] CONRAD M. E., CROSBY W. H. (1960). Massive nitrogen mustard therapy in Hodgkin's disease with protection of bone marrow by tourniquets.. Blood.

[OCR_01545] DUNJIC A., MAISIN J. (1960). [Curative action of isologous bone marrow in rats having received a lethal dose of myleran. Relation between the number of cells of transfused bone marrow and survival].. Rev Fr Etud Clin Biol.

[OCR_01549] HUMBLE J. G., NEWTON K. A. (1958). Technique of human bone-marrow transplants.. Lancet.

[OCR_01555] JACOBSON L. O., SIMMONS E. L., MARKS E. K., ROBSON M. J., BETHARD W. F., GASTON E. O. (1950). The role of the spleen in radiation injury and recovery.. J Lab Clin Med.

[OCR_01563] JAMMET H., MATHE G., PENDIC B., DUPLAN J. F., MAUPIN B., LATARJET R., KALIC D., SCHWARZENBERG L., DJUKIC Z., VIGNE J. (1959). Etude de six cas d'irradiation totale aiguë accidentelle.. Rev Fr Etud Clin Biol.

[OCR_01569] LAJTHA L. G. (1960). Consideration of the theory of bone marrow grafting as treatment of radiation damage.. Br J Radiol.

[OCR_01571] LORENZ E., CONGDON C., UPHOFF D. (1952). Modification of acute irradiation injury in mice and guinea-pigs by bone marrow injections.. Radiology.

[OCR_01579] MATHE G., BERNARD J., de VRIES M., SCHWARZENBERG L., LARRIEU M. J., LALANNE C. M., DUTREIX A., AMIEL J. L., SURMONT J. (1960). [New trials with homologous bone marrow grafts after total irradiation in children with acute leukemia in remission. The problem of the secondary syndrome in man].. Rev Hematol.

[OCR_01582] MATHE G., JAMMET H., PENDIC B., SCHWARZENBERG L., DUPLAN J. F., MAUPIN B., LATARJET R., LARRIEU M. J., KALIC D., DJUKIC Z. (1959). Transfusions et greffes de moelle osseuse homologue chez des humains irradiés à haute dose accidentellement.. Rev Fr Etud Clin Biol.

[OCR_01598] SELLEI C., ECKHARDT S. (1958). Clinical observations with 1, 6-bis(beta-chloroethylamino)-1, 6-deoxy-D-mannitol dihydrochloride (BCM) in malignant diseases.. Ann N Y Acad Sci.

[OCR_01612] SYKES M. P., CHU F. C., WILKERSON W. G. (1960). Local bone-marrow changes secondary to therapeutic irradiation.. Radiology.

[OCR_01600] Smiley R. K., Martin-Villar J., Belanger L. F. (1961). Effect of Autologous Bone Marrow on the Cytopenias Induced by Nitrogen Mustard.. Can Med Assoc J.

[OCR_01610] Stoll B. A., Matar J. H. (1961). Cyclophosphamide in Advanced Breast Cancer.. Br Med J.

[OCR_01618] TALBOT T. R., ELSON L. A. (1958). Protection of August rats against lethal doses of a dimethyl homologue of myleran by isologous bone marrow.. Nature.

[OCR_01622] TUBIANA M., BERNARD C. I., LALANNE C. (1959). [Modification of erythropoiesis after pelvic radiotherapy].. Acta radiol.

